# HNRNPA1-mediated 3′ UTR length changes of *HN1* contributes to cancer- and senescence-associated phenotypes

**DOI:** 10.18632/aging.102060

**Published:** 2019-06-30

**Authors:** Qi Jia, Hongbo Nie, Peng Yu, Baiyun Xie, Chenji Wang, Fu Yang, Gang Wei, Ting Ni

**Affiliations:** 1 State Key Laboratory of Genetic Engineering, Collaborative Innovation Center of Genetics and Development, Human Phenome Institute, School of Life Sciences, Fudan University, Shanghai 200438, P.R. China; 2 Department of Biology, Southern University of Science and Technology, Shenzhen, Guangdong 518055, P.R. China; 3 State Key Laboratory of Genetic Engineering, Collaborative Innovation Center of Genetics and Development, School of Life Sciences, Fudan University, Shanghai 200438, P.R. China; 4 Department of Medical Genetics, Second Military Medical University, Shanghai 200433, P.R. China

**Keywords:** cancer, senescence, alternative polyadenylation, *HN1*, HNRNPA1

## Abstract

Cellular senescence has been regarded as a mechanism of tumor suppression. Studying the regulation of gene expression at various levels in cell senescence will shed light on cancer therapy. Alternative polyadenylation (APA) regulates gene expression by altering 3′ untranslated regions (3′ UTR) and plays important roles in diverse biological processes. However, whether APA of a specific gene functions in both cancer and senescence remains unclear. Here, we discovered that 3′ UTR of *HN1* (or *JPT1*) showed shortening in cancers and lengthening in senescence, correlated well with its high expression in cancer cells and low expression in senescent cells, respectively. *HN1* transcripts with longer 3′ UTR were less stable and produced less protein. Down-regulation of *HN1* induced senescence-associated phenotypes in both normal and cancer cells. Patients with higher *HN1* expression had lower survival rates in various carcinomas. Interestingly, down-regulating the splicing factor HNRNPA1 induced 3′ UTR lengthening of *HN1* and senescence-associated phenotypes, which could be partially reversed by overexpressing *HN1*. Together, we revealed for the first time that HNRNPA1-mediated APA of *HN1* contributed to cancer- and senescence-related phenotypes. Given senescence is a cancer prevention mechanism, our discovery indicates the HNRNPA1-*HN1* axis as a potential target for cancer treatment.

## INTRODUCTION

Alternative polyadenylation (APA) of mRNA is a prevalent post-transcriptional gene regulation mechanism in eukaryotes [1]. APA can be divided into UTR-APA (untranslated region APA) and CR-APA (coding region APA), based on the location of polyadenylation (pA) sites [2]. UTR-APA produces transcripts with 3′ UTRs of different length, affecting mRNA stability, translational efficiency, and the cellular localization of mRNA and protein [3], which largely depend on the interactions between alternative 3′ UTR (sequences between the proximal and distal pA sites) and other molecules such as microRNAs (miRNAs), RNA-binding proteins (RBPs) and/or long noncoding RNAs (lncRNAs) [4, 5]. Many RNA binding proteins, including those involved in mRNA 3′ end formation and alternative splicing, can modulate the alternative pA site usage, indicating polyadenylation and splicing could be interrelated [6]. For example, SRSF3 (or SRp20), one member of the serine/arginine-rich (SR) splicing factor family, can regulate the alternative pA site recognition of calcitonin coding gene *CALCA* [7]. HNRNPC, a member of heterogeneous nuclear ribonucleoproteins (HNRNP) family that involved in alternative splicing, has a genome-wide effect on APA regulation of mRNA with poly(U) motifs [8]. Intriguingly, many SR and HNRNP splicing factors are abnormally expressed in a variety of cancer types [9]. However, whether they could function in regulating APA and cancer-associated processes remains to be explored.

APA-mediated global shortening of 3′ UTRs is prevalent in cancer [10]. 3′ UTR shortening of some gene, such as *IMP-1*, can even contribute to cancer development. Cancer cells can be regarded as a state escaping from cellular senescence [11], and cellular senescence can serve as an anti-cancer mechanism in both young and elders [12]. Therefore, inducing cancer cells to enter into senescent state is a promising cancer immunotherapy strategy [13]. Cancer and senescence have opposite characteristics in many aspects, such as proliferation capacity and expression of typical molecular markers [14, 15]. Interestingly, our recent publication demonstrated that cell senescence underwent 3′ UTR lengthening, and longer 3′ UTR of a specific gene, *Rras2*, contributed to senescence-associated phenotypes [16]. These above evidences provide the clues that APA-mediated 3′ UTR length regulation may have the possibility to switch cell fate between cancer and senescence. However, whether a single gene could regulate such cell fate transition remains to be known.

To explore whether APA of a specific gene could contribute to cancer or senescence and the possible upstream regulators, we performed an integrative analysis on multiple data sets of cancer and senescence. *HN1* *(Hematopoietic- and neurologic-expressed sequence 1)* was finally screened out due to its preferring 3′ UTR shortening in multiple tumor tissues and favoring longer 3′ UTRs in diverse senescent cells [16, 17]. *HN1* transcript with longer 3′ UTR was less stable and resulted in decreased protein abundance compared with transcript with shorter 3′ UTR. Downregulation of *HN1* caused senescence-associated phenotypes in both regular and cancer cells. Further investigation revealed HNRNPA1 (Heterogeneous Nuclear Ribonucleoprotein A1) as a novel regulator of APA in *HN1*, increased *HNRNPA1* expression is responsible for 3′ UTR shortening, while decreased *HNRNPA1* is responsible for 3′ UTR lengthening. This study uncovered for the first time that HNRNPA1-mediated 3′ UTR length changes of *HN1* could contribute to cancer- and senescence-associated phenotypes, providing a new perspective to understand the molecular events underlying cancer and senescence, and indicating a potential target for cancer treatment as well.

## RESULTS

### APA-mediated 3′ UTR shortening of *HN1* in carcinomas and lengthening in senescence

**Figure 1:**
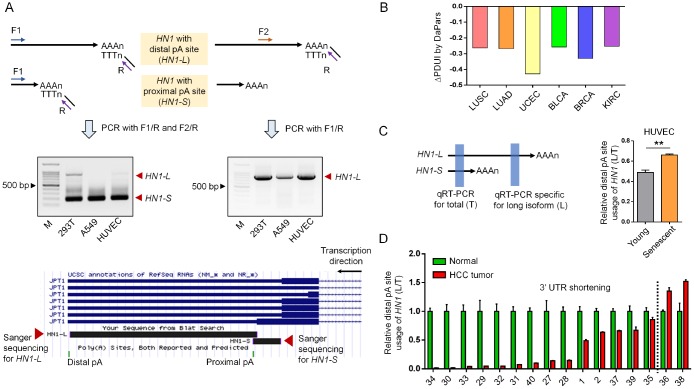
**APA-mediated 3′ UTR length changes of *HN1* in cancer and senescent cells.** (**A**) 3′ RACE assay to validate *HN1* (also known as *JPT1*) isoforms with different 3′ UTR length in HEK293T, A549, and HUVEC cells. Forward (F) and Reverse (R) primer pairs used in the 3′ RACE were illustrated (top panel), followed by the gel image showing the PCR fragments representing two isoforms with different 3′ UTR length (middle panel). 3′ RACE amplicon corresponding to either distal or proximal pA site was Sanger sequenced and mapped to human genome (hg38), as was illustrated by UCSC Genome Browser (bottom panel). (**B**) APA-mediated 3′ UTR shortening of *HN1* in six cancer types comparing to matched normal tissues based on public data [17]. Y axis stands for the ΔPDUI value (change in Percentage of Distal polyA site Usage Index) quantified by DaPars method. A minus ΔPDUI value represents 3′ UTR shortening. (**C**) qRT-PCR assay to evaluate the usage of distal pA site (L) compared to the total isoform expression (T) of *HN1* among young (passage 6) and senescent (passage 15) HUVECs (**, *t*-test, p < 0.01). Left panel showed diagram of the primer pair design, as highlighted in blue rectangle. Right panel showed qRT-PCR result. (**D**) The relative expression of long isoform normalized to total *HN1* expression (L/T) was measured by qRT-PCR assay among 16 paired samples of hepatocellular carcinoma (HCC). The numbers in the X axis represent the labeling ID of given patients. Left part to the dashed black line represented patients with 3′ UTR shortening of *HN1*.

By comparing genes preferring shorter 3′ UTR in seven cancer types based on RNA-seq (RNA sequencing) datasets from TCGA (The Cancer Genome Atlas) and genes preferring longer 3′ UTR in senescent mouse embryonic fibroblasts (MEFs) and rat vascular smooth muscle cells (rVSMC) based on PA-seq (polyadenylation sequencing) [16, 17], we screened out 36 genes exhibiting opposite changes in 3′ UTR length between cancer and senescence. From these, *HN1* (also known as *JPT1*) was selected for studying the functional link between APA and these two opposite biological processes, since it has been reported to promote tumorigenesis [18, 19]. The APA of *HN1* was cross-validated by two methods capable of capturing precise pA sites via globally sequencing the 3′ end of mRNAs (PolyA-Seq and 3′ READS (3′ region extraction and deep sequencing) [20, 21]. What’s more, two pA sites clearly appeared in *HN1* by track search on multiple human cells and tissues through UCSC Genome Browser (Supplementary Figure 1). Finally, these two pA sites were experimentally validated in three human cell lines including human embryonic kidney cells (HEK293T), human umbilical vein endothelial cells (HUVEC), and human lung adenocarcinoma epithelial cells (A549) using rapid amplification of cDNA 3′ end (3′ RACE) assay and Sanger sequencing (Figure 1A). These results demonstrated UTR-APA really existed in *HN1*.

Since UTR-APA produces isoforms with different 3′ UTR length, we next examined whether *HN1*’s 3′ UTR was dynamically changed between cancer and senescence. By evaluating 3′ UTR length changes with the ΔPDUI (Percentage of Distal polyA site Usage Index) [17], we found *HN1* prefered the proximal pA site in six out of seven tested cancers, such as LUSC (Lung squamous cell carcinoma), LUAD (Lung adenocarcinoma), UCEC (Uterine Corpus Endometrial Carcinoma), BLCA (Bladder Urothelial Carcinoma), BRCA (Breast invasive carcinoma), and KIRC (Kidney renal clear cell carcinoma), indicating *HN1* underwent a general 3′ UTR shortening in cancer (Figure 1B). In contrast, 3′ UTR lengthening of *HN1* was discovered in senescent HUVECs, rVSMCs and MEFs by our PA-seq method (Supplementary Figures 2–4) and was validated in senescent HUVEC cells by reverse transcription and quantitative polymerase chain reaction (qRT-PCR) using primers for common or alternative 3′ UTR (Figure 1C). In addition, qRT-PCR result of 16 hepatocellular carcinoma (HCC) cases from Chinese population also showed that the shorter 3′ UTR of *HN1* was preferred in most tumor samples compared to matched normal tissues (Figure 1D). The above findings collectively demonstrated that *HN1* underwent 3′ UTR shortening in various human cancers and 3′ UTR lengthening during cellular senescence.

### *HN1* shows opposite expression trends between cancer and senescence

**Figure 2:**
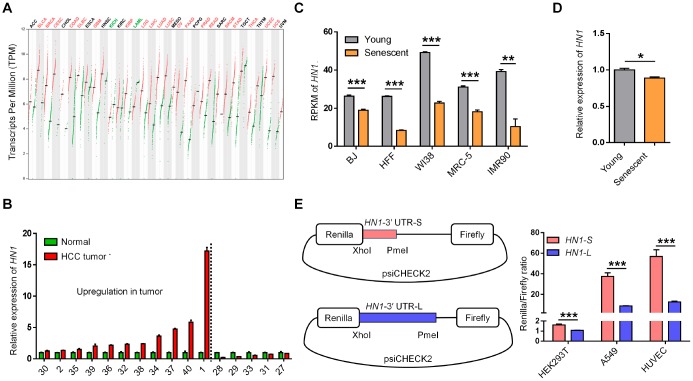
**The opposite expression pattern of *HN1* between cancer and senescence.** (**A**) *HN1* mRNA expression in various cancer types based on GEPIA [23]. The red line and green line within a rectangle represent tumor tissues and normal tissues, respectively. Median expression values were denoted with the crossed black short line. Significantly increased and decreased expressions in tumor comparing to matched normal tissue indicated as red and green fonts at the top, respectively. (**B**) qRT-PCR measured *HN1* expression levels in paired normal and tumor tissues of 16 HCC patients. The numbers on X axis represents the same labeling ID of a patient as described in Figure 1D. Left part to dashed line represented patients with upregulation of *HN1*. (**C**) *HN1* expression in various senescent cells based on public RNA-seq datasets [24], indicated by RPKM (reads per kilobase per million reads). Lower *HN1* expression was observed in five human senescent models (error bars and *t*-test were based on three biological replicates. **, p < 0.01; ***, p < 0.001). (**D**) *HN1* expression changes between young (passage 6) and senescent (passage 15) HUVECs evaluated by qRT-PCR. * stands for p < 0.05 (*t*-test with three PCR replicates). (**E**) Dual-luciferase reporter assay to test the influence of 3′ UTRs (*HN1-S* and *HN1-L*) on protein production in HEK293T, A549, and HUVEC cells. Relative luminescence of Renilla luciferase was normalized using the reference Firefly luciferase activity, as shown in the left panel. *** represents a p value less than 0.001 in *t*-test with four biological replicates.

To examine the potential consequences of 3′ UTR length changes in cancer and senescence, we first quantified the steady-state mRNA levels, as APA-mediated 3′ UTR length changes could affect mRNA decay [1, 22]. By analyzing RNA-seq data from TCGA database [23], we observed higher expression of *HN1* in 20 cancer types compared to normal tissues (Figure 2A). *HN1* was also highly expressed in most Chinese HCC patients compared to matched controls (Figure 2B). Importantly, individuals with high *HN1* expression tend to have lower survival rates in various cancer types, such as KIRP (Kidney renal papillary cell carcinoma), LIHC (Liver hepatocellular carcinoma), ACC (Adrenocortical carcinoma), HNSC (Head and Neck squamous cell carcinoma), KIRC, PAAD (Pancreatic adenocarcinoma), SKCM (Skin Cutaneous Melanoma), LUAD, and LUSC (Supplementary Figure 5), indicating that *HN1* may function in tumor progression. Since cellular senescence can be considered as a cancer prevention mechanism [13, 24], changes in *HN1* expression were then analyzed in multiple senescence models. Interestingly, *HN1* showed decreased expression in multiple senescent cells, including BJ fibroblasts, human foreskin fibroblasts (HFF), three human embryonic lung fibroblasts (WI-38, MRC-5, and IMR90), and HUVEC cells (Figure 2C and 2D). These results indicated that *HN1* tended to express oppositely between cancer and senescence.

Compared to shorter 3′ UTRs in transcripts, longer 3′ UTRs are likely to introduce more recognition sites for miRNAs and/or RBPs, which could affect mRNA stability and/or translational efficiency [1, 25]. We then investigated the downstream effects of the two transcripts of *HN1* with shorter 3′ UTR (*HN1-S*) and longer 3′ UTR (*HN1-L*) on gene expression, respectively. Interestingly, *HN1-L* was found to produce less protein than *HN1-S* in all three tested human cell lines (293T, A549, and HUVEC), as evaluated by dual-luciferase reporter assay (Figure 2E), suggesting 3′ UTR lengthening of *HN1* contributes to reduced protein abundance.

### Knockdown of *HN1* induces cellular senescence in both normal and cancer cells

**Figure 3:**
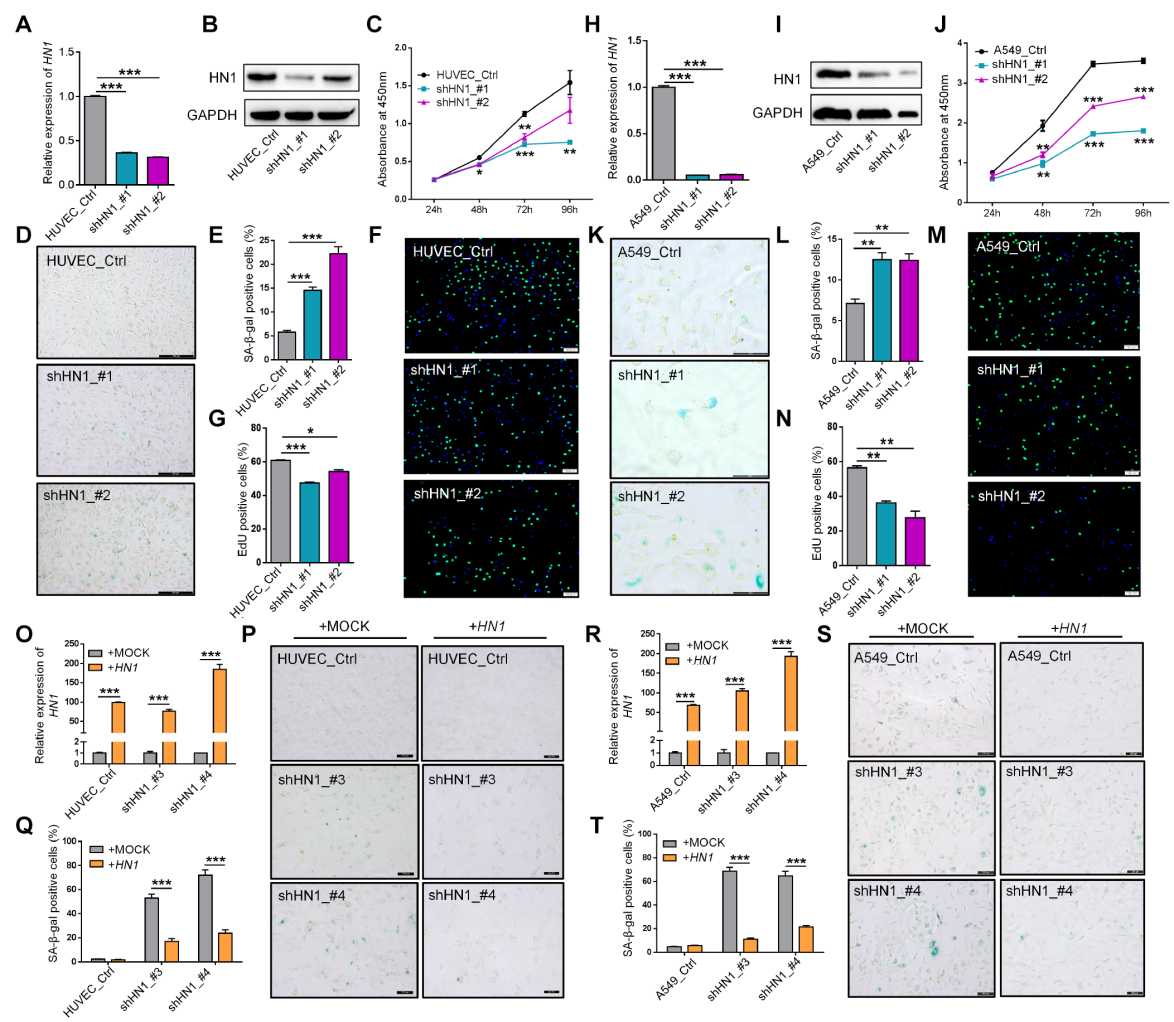
**Knockdown of *HN1* induces cellular senescence in normal and cancer cells.** (**A**–**B**) Validation of *HN1* knockdown (KD) in HUVEC with two shRNAs (shHN1_#1, shHN1_#2), as quantified by qRT-PCR (**A**) and Western blot (**B**), respectively. *GAPDH* served as internal control for both mRNA and protein. (**C**) Cell proliferation rate evaluated by Cell Counting Kit-8 (CCK-8) assay in *HN1*-KD HUVEC cells with two shRNAs. *, ** and *** stand for p < 0.05, p < 0.01 and p < 0.001, respectively, based on *t*-test with three biological replicates. (**D**–**E**) Representative SA-β-Gal staining (D) and quantitative statistics (E) in *HN1*-KD and control HUVEC cells. *** represents p < 0.001 based on *t*-test with three independent countings. (**F**–**G**) EdU incorporation assay (**F**) and quantitative statistics (**G**) in *HN1*-KD and control HUVEC cells. Green and blue dots stand for incorporated EdU and DNA DAPI (4',6-diamidino-2-phenylindole) staining, respectively. * and *** stand for p < 0.05 and p < 0.001, respectively, based on *t*-test with three independent countings. (**H**–**I**) Validation of *HN1* knockdown in A549 cells, as described in panel A-B. (**J**) Cell proliferation rate evaluated by CCK-8 assay in *HN1*-KD A549 cells, as described in panel C. (**K**–**L**) SA-β-Gal staining (**K**) and quantitative statistics (**L**) were shown in A549 cells, as described in panel D-E. ** represents p < 0.01 based on *t*-test with three independent countings. (**M**–**N**) EdU incorporation assay (**M**) and positive cell (EdU incorporated) statistics (**N**) were shown in A549 cells, as described in panel F-G. ** represents p < 0.01 based on *t*-test with three independent countings. (**O**–**Q**) Over-expression of *HN1* partially rescued *HN1*-KD induced SA-β-Gal activity in HUVECs. qRT-PCR confirmed overexpression of *HN1* in *HN1*-KD HUVECs (**O**). *** represents p < 0.001 based on *t*-test with three qPCR reactions. SA-β-Gal staining (**P**), and positive staining cell statistics (**Q**) in control (+MOCK) and overexpression (+*HN1*) HUVECs. *** represents p < 0.001 based on *t*-test with three independent countings. (**R**–**T**) Over-expression of *HN1* partially rescued *HN1*-KD induced SA-β-Gal activity in A549 cells. qRT-PCR confirmed overexpression of *HN1* in *HN1*-KD A549 cells (**R**). SA-β-Gal staining (**S**) and positive staining cell statistics (**T**) in control (+MOCK) and overexpression (+*HN1*) A549 cells. *** represents p < 0.001, as described in panel O-Q.

Given 3′ UTR lengthening of *HN1* contributed to the down-regulation of protein production, we knocked down the expression of *HN1* in both normal cells (293T, and HUVEC) and cancer cells (A549) to mimic the consequence of 3′ UTR lengthening. Lentiviral-mediated *HN1* knockdown (KD) through two short hairpin RNAs (shHN1_#1, shHN1_#2) was confirmed by both qRT-PCR and Western blot in HUVEC (Figure 3A and 3B), A549 (Figure 3H and 3I), and 293T cells (Supplementary Figure 7A and 7B). Interestingly, a series of senescence-associated cellular phenotypes emerged when *HN1* was stably knocked down. First, all three cell lines showed decreased proliferation rate (Figure 3C, 3J and Supplementary Figure 7C) and increased percentage of senescence-associated SA-β-Gal positive cells (Figure 3D–3E, 3K–3L, Supplementary Figure 7D and 7E) [26, 27]. Second, decreased DNA synthesis rate was observed in *HN1*-KD cells by the EdU incorporation assay (Figure 3F–3G, 3M–3N), which could be regarded as another indicator for senescence [28]. Next, *HN1*-KD induced G2/M cell cycle arrest in HUVEC cells and G1 arrest in A549 cells (Supplementary Figure 6A–6B; 6F–6G), in addition to a significantly decreased proportion of S-phase cells in both *HN1*-KD cell lines (Supplementary Figure 6A–6B; 6F–6G). *LMNB1,* a senescence marker indicating nuclear changes of senescent cells [29], showed significantly decreased expression in both *HN1*-KD HUVECs and A549 cells (Supplementary Figure 6C, 6H). What’s more, the level of intracellular reactive oxygen species (ROS), a known senescence-associated phenotype and also a contributor to senescence [30], slightly increased in *HN1*-KD cells (Supplementary Figure 6D, 6I). Finally, several other senescence-associated molecular markers, including *CDK1*, *CCNB1,* and *IL6* [31], were also used to evaluate the senescence in *HN1*-KD cells. The result showed that *HN1*-KD induced down-regulation of *CDK1*, *CCNB1* and up-regulation of *IL6* in both HUVECs and A549 cells (Supplementary Figure 6E, 6J). These above combined to support the notion that *HN1*-KD could induce senescence-associated phenotypes in both normal and cancer cells.

Since *HN1*-KD could induce cellular senescence in a variety of cell types, we then went on to test whether this senescent state was *HN1*-dependent or not. Interestingly, overexpression of *HN1* in *HN1*-KD HUVECs and HEK293T cells could alleviate the senescence state to some extent (Figure 3O–3Q, Supplementary Figure 7F–7H). Similar results were also observed in A549 cancer cell line (Figure 3R–3T). This further confirms that *HN1* can act as a regulator of cellular senescence.

### The 3′ UTR length of *HN1* was regulated by HNRNPA1

**Figure 4:**
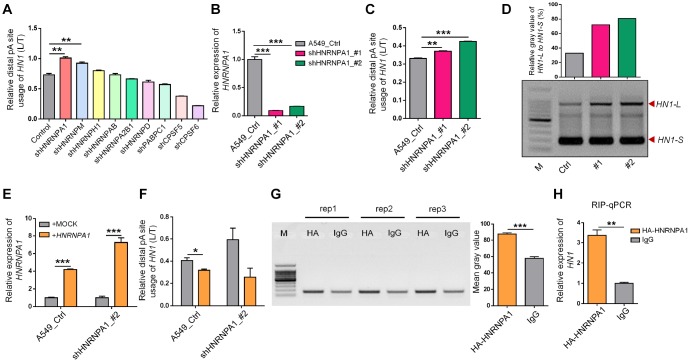
**HNRNPA1 binds to mRNA of *HN1* and regulates its 3′ UTR length changes.** (**A**) The relative *HN1-L* expression to total mRNA levels (L/T) evaluated by qRT-PCR upon knockdown of various genes encoding candidate RNA binding proteins in HEK293T. ** represents p < 0.01 based on *t*-test with three qPCR reactions. (**B**) Knockdown of *HNRNPA1* by two shRNAs (shHNRNPA1_#1, shHNRNPA1_#2) was confirmed by qRT-PCR in A549 cells. *** represents p < 0.001 based on *t*-test with three qPCR reactions. (**C**) Higher usage of *HN1-L* to total *HN1* mRNA (L/T) upon *HNRNPA1* knockdown in A549 cells was evaluated by qRT-PCR analysis. ** and *** represent p < 0.01 and p < 0.001, respectively, based on *t*-test with three qPCR reactions. (D) 3′ RACE assay showed higher abundance of *HN1-L* compared to *HN1-S* upon knockdown of *HNRNPA1*. Relative mean gray value of *HN1-L* compared with *HN1-S* was shown at the upper panel by ImageJ. (E-F) *HNRNPA1* overexpression reversed *HNRNPA1*-KD induced 3′ UTR lengthening of *HN1.* Overexpression of *HNRNPA1* was confirmed in both *HNRNPA1*-KD and control A549 cells (E). Decreased usage of *HN1-L* compared to total *HN1* mRNA (L/T) was detected by qRT-PCR upon overexpression of *HNRNPA1* in A549 cell (F). * and *** represent p < 0.05 and p < 0.001, respectively, based on *t*-test with three qPCR reactions. (G) RNA binding protein immunoprecipitation coupled with PCR (RIP-PCR) was performed in A549 cells transfected with the HA-tagged *HNRNPA1*-overexpression plasmid. Immunoprecipitated RNA with either HA-antibody or control IgG was reversely transcribed and amplified with primer pairs specific for *HN1* mRNA. Agarose gel of three independent RIP-PCR experiments (rep1, 2, 3) was shown at left and mean gray value was shown at right. *** represents p < 0.001 based on *t*-test with three RIP-PCR reactions at the left. (H) Quantitative PCR for immunoprecipitated RNA (RIP-qPCR) to test the enrichment of *HN1 mRNA* in HA-antibody compared to control IgG in A549 cells. ** represents p < 0.01 based on *t*-test with three qPCR reactions.

We next searched the upstream regulators responsible for APA-mediated 3′ UTR length changes in *HN1.* Since RBPs were widely reported to regulate alternative polyadenylation of certain genes [32], we tried to narrow down the potential RBPs with the following criteria: 1) have potential binding motifs located at the 3′ UTR; 2) have relatively high expression in HUVEC, 293T and A549 cells; 3) opposite expression trends between cancer and senescence; 4) with the reported function to regulate either alternative polyadenylation (APA) or alternative splicing (AS) due to the crosstalk between APA and AS. Nine candidate RBPs (HNRNPA1, HNRNPAB, HNRNPA2B1, HNRNPH1, HNRNPD, HNRNPM, CPSF5, CPSF6, and PABPC1) were screened out to examine their effect on the relative abundance of *HN1* transcripts with alternative 3′ UTR caused by APA. Knockdown of HNRNPA1 or HNRNPM could promote the relative expression of *HN1-L* compared to total expression (*HN1-L* and *HN1-S*, labelled as *HN1-T*), but HNRNPA1 demonstrated the maximun effect (Figure 4A), so HNRNPA1 was choosed for further investigation. The ability of *HNRNPA1* to regulate APA in *HN1* was further validated separately in *HNRNPA1*-KD HEK293T cells (Supplementary Figure 8C–8D), A549 cells (Figure 4B–4D) and HUVEC cells (Supplementary Figure 8A, 8B). Furthermore, the increased L/T ratio in *HNRNPA1*-KD cells could be reversed with *HNRNPA1* overexpression (Figure 4E, 4F, Supplementary Figure 8E and 8F). These above results gave the strong evidences that splicing factor HNRNPA1 could promote the relative selection of proximal pA site in *HN1*, thereby adjusting the corresponding 3′ UTR length.

To further test whether HNRNPA1 could bind to mRNA of *HN1*, RNA immunoprecipitation coupled with reverse transcription PCR (RIP-PCR) was performed in cells expressing HA-tagged HNRNPA1. RNAs interacting with HNRNPA1 were pulled down with HA antibody and negative control IgG, then the level of *HN1* mRNA was quantified by both RT-PCR and qRT-PCR. The result showed that HNRNPA1 was highly enriched on mRNA of *HN1* compared to control samples in all three tested human cells (Figure 4G, 4H, Supplementary Figure 8G–8J), while a non-relevant mRNA of *GAPDH* had no such enrichment (Supplementary Figure 8K–8M), indicating that HNRNPA1 bound directly to mRNA of *HN1* to inhibit the distal pA site selection.

### Down-regulation of *HNRNPA1* promotes senescence-associated phenotypes through reduced *HN1* expression

**Figure 5:**
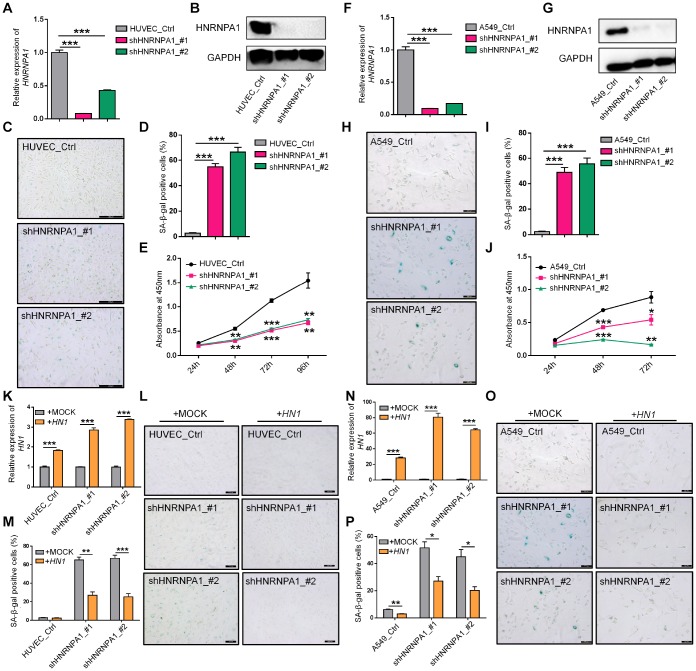
***HNRNPA1* knockdown induces senescence-associated phenotypes and *HN1* overexpression rescues *HNRNPA1*-KD induced SA-β-Gal activity.** (**A**–**E**) Senescence-associated phenotypes were detected in *HNRNPA1*-KD HUVECs. *HNRNPA1* knockdown by two shRNAs was validated by both qRT-PCR (**A**) and Western blot (**B**). *** represents p < 0.001 based on *t*-test with three qPCR reactions. SA-β-Gal staining positive cells increased (**C**, **D**) and cell proliferation rate decreased (evaluated by CCK-8 assay) (**E**) in *HNRNPA1*-KD cells. *** in D represents p < 0.001 based on *t*-test with three independent countings. ** and *** in E stand for p < 0.01 and p < 0.001, respectively, based on *t*-test with three biological replicates. (**F**–**J**) The same assays in panels A-E were performed in *HNRNPA1*-KD A549 cells, including mRNA levels (**F**), protein levels (**G**), SA-β-Gal staining (**H**, **I**), and CCK-8 assay (**J**), as described in panel A-E. *, ** and *** in J stand for p < 0.05, p < 0.01 and p < 0.001, respectively, based on *t*-test with three biological replicates. (**K**–**M**) Overexpression of *HN1* reversed *HNRNPA1*-KD induced SA-β-Gal staining in HUVECs. Overexpression of *HN1* was confirmed in both *HNRNPA1*-KD and control HUVEC cells (**K**). *** represents p < 0.001 based on *t*-test with three qPCR reactions. Representative SA-β-Gal staining (**L**) and staining-positive cell statistics (**M**) in control (+MOCK) and *HN1* overexpression (+*HN1*) HUVECs were shown. ** and *** in M stand for p < 0.01 and p < 0.001, respectively, based on *t*-test with three independent countings.(**N**–**P**) Overexpression of *HN1* reversed *HNRNPA1*-KD induced SA-β-Gal staining in A549 cells. Overexpression of *HN1* was confirmed in both *HNRNPA1*-KD and control A549 cells (**N**). *** represents p < 0.001 based on *t*-test with three qPCR reactions. Representative SA-β-Gal staining (**O**) and staining-positive cell statistics (**P**) in control (+MOCK) and overexpression (+*HN1*) A549 cells were shown. * and ** in P stand for p < 0.05 and p < 0.01, respectively, based on *t*-test with three independent countings.

Since *HNRNPA1*-KD resulted in the higher proportion of *HN1-L*, which produced less protein and gave rise to senescence-related phenotypes, one would expect that down-regulation of *HNRNPA1* could also induce cellular senescence. To test this hypothesis, we knocked down *HNRNPA1* in HUVEC and A549 cells (Figure 5A, 5B, 5F and 5G), and examined some senescence-associated phenotypes. Interestingly, *HNRNPA1*-KD led to a higher percentage of positive SA-β-Gal stained cells and slower cell growth rate in both cell types (Figure 5C–5E, 5H–5J). Besides, *HNRNPA1*-KD HUVECs and A549 cells also showed arrested G2/M phase, decreased *LMNB1* expression, increased ROS production, and altered expression of senescence-associated molecular markers (including *CDK1*, *CDK2*, *CCNB1*, and *IL6*) (Supplementary Figure 9A–9J), resembling to the effects caused by *HN1*-KD. To investigate whether *HNRNPA1*-KD-induced senescence was dependent on decreased *HN1* expression, we overexpressed *HN1* in *HNRNPA1*-KD cells, and found that most of the SA-β-Gal staining was reversed (Figure 5K–5M, 5N–5P). These above results strongly suggest that APA-mediated reduction of HN1 protein contributes, at least in part, to *HNRNPA1*-KD induced cellular senescence.

### Down-regulation of *HN1* and *HNRNPA1* inhibits cancer-related phenotypes

**Figure 6:**
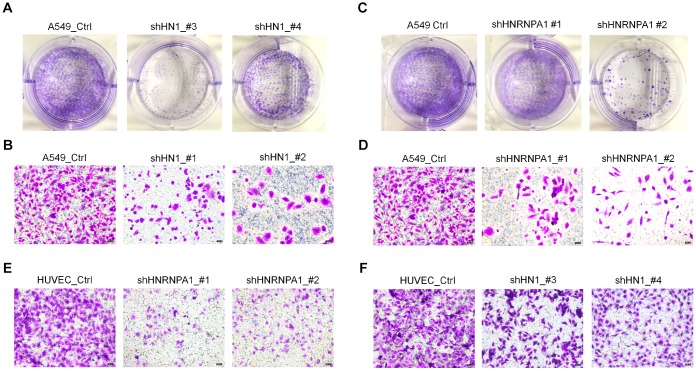
**Knockdown of *HN1* and *HNRNPA1* inhibits ability of colony formation and cell migration.** (**A**) The colony formation capacity between control (Ctrl) and *HN1*-KD (two shRNAs) A549 cells was detected by crystal violet staining. (**B**) Cell migration ability in *HN1*-KD (two shRNAs) A549 cells was detected by transwell assay. (**C**–**D**) The ability of colony formation (**C**) and cell migration (**D**) was weakened in *HNRNPA1*-KD (two shRNAs) A549 cells. (**E**–**F**) Cell migration ability was weakened in *HNRNPA1*-KD (**E**, two shRNAs) and *HN1*-KD HUVECs (**F**, two shRNAs) when comparing to control cells, as evaluated by transwell migration assay.

The fact that cancer cells exhibited 3′ UTR shortening of *HN1* and up-regulation of *HNRNPA1* while senescent cells displayed 3′ UTR lengthening of *HN1* and down-regulation of *HNRNPA1* (Supplementary Figure 10A–10D) can be well explained by the HNRNPA1-mediated APA regulation of *HN1*. These results prompted us to hypothesize that HNRNPA1-mediated changes in *HN1* expression not only can regulate cellular senescence but also have the potential to regulate cancer-associated phenotypes. To test this, we performed colony formation and cell migration assays in the lung cancer cell line A549 before and after knocking down *HN1* or *HNRNPA1*. Interestingly, *HN1* deficiency reduced the colony formation efficiency (Figure 6A) and cell migration ability (Figure 6B, Supplementary Figure 11A). Coincidentally, knockdown of *HNRNPA1* in A549 cells also resulted in similar phenotypes as that in *HN1*-KD cells (Figure 6C and 6D, Supplementary Figure 11B). Moreover, *HN1*-KD and *HNRNPA1*-KD also showed decreased cell migration rate in HUVEC cells (Figure 6E and 6F, Supplementary Figure 11C–11D). Consistent with the previous observation that patients with higher *HN1* expression have worse survival prognosis (Supplementary Figure 5), higher *HNRNPA1* expression was also associated with lower survival rates in multiple cancer types (Supplementary Figure 12). Altogether, these results suggested that down-regulation of *HN1* and *HNRNPA1* could inhibit cancer-related cell phenotypes and contribute to patient survival.

## DISCUSSION

**Figure 7:**
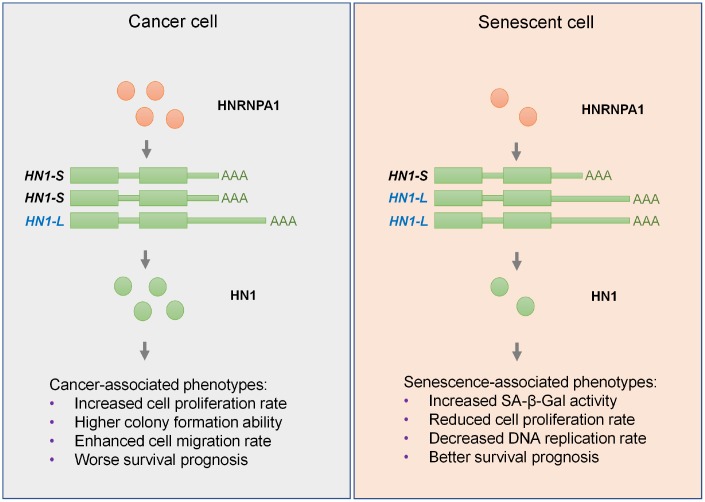
**Graphic abstract for HNRNPA1-mediated 3′ UTR length changes contributes to cancer- and senescence-associated phenotypes.** In cancer cells, upregulated HNRNPA1 leads to higher usage of proximal pA site of *HN1* and thus increases the level of its transcripts with shorter 3′ UTR, which produces more protein than the longer one, and ultimately promotes cancer-associated phenotypes. In senescent cells, HNRNPA1 is downregulated and causes higher usage of distal pA site of *HN1*. Such regulation lengthens the 3′ UTR of *HN1* and generates less protein, which in turn promotes senescence-associated phenotypes in both normal and cancer cells.

Our recent work indicates that 3′ UTR lengthening is a novel mechanism in regulating cell senescence [16]. However, whether a single gene shows 3′ UTR lengthening in senescent cells and 3′ UTR shortening in cancer cells can function in cell fate decision is unknown. By combining integrative analyses and experimental validations, we screened out *HN1*, whose UTR-APA demonstrate regulatory roles in both senescence and cancer. We further showed that *HNRNPA1* expression increased in cancer and decreased in senescence well explained the related APA-mediated 3′ UTR length changes in *HN1*. The discovery that HNRNPA1-mediated 3′ UTR length changes in *HN1* contributed to cancer- and senescence-associated phenotypes largely expands our knowledge in post-transcriptional regulation in cancer and senescence (Figure 7).

*HN1* has been reported to have important biological functions. *HN1* was conservatively expressed in multiple species [33], and was known to be associated with neural development [34], retina regeneration [35], and tumor progression [36]. *HN1* could promote breast cancer progression by increasing MYC activity [37], and contribute to cell growth and migration in prostate cancer [18]. What’s more, *HN1* plays an important role in androgen-receptor mediated signaling and promotes proteosomal degradation of androgen-receptor in prostate cell lines [38]. In our study, we found *HN1* could also paly a role in senescence-asscociated phenotypes (Figure 3) in addition to apoptosis and necrosis in HUVECs and A549 cells (Supplementary Figure 13).

Although miRNAs and/or RBPs located in alternative 3′ UTR can explain the differences in RNA or protein levels of their target genes [39], how they regulate *HN1*’s expression at the post-transcriptional level deserves further study. Though transcripts with longer 3′ UTR (*HN1-L*) were well validated to generate less protein than the shorter ones (Figure 2E), whether the reduced protein level is attributed to faster RNA degradation or weakened translational efficiency was unclear. To address this question, we performed RNA stability assay and found that *HN1-L* had a slightly faster mRNA degradation rate compared to *HN1-S* in 293T cells (Supplementary Figure 14), indicating that in addition to RNA stability, translational regulation may also explain the reduced protein level for this situation. Interestitly, alternative 3′ UTR of *HN1* can be recognized by miR-132 to repress its translation [19]. Noteworthy, various miRNAs and RBPs with the potential to bind to the alternative 3′ UTR of *HN1* were predicted with TargetScan [40] (Supplementary Figure 15) and RBPmap [41] (Supplementary Figure 16), respectively. Further experimental screening is needed to figure out detailed regulators responsible for the reduced protein production of *HN1-L*.

In the present study, we first explored the correlation between the changes of 3′ UTR length and expression of *HN1* through the published next-generation sequencing data in cancer [17]. *HN1*’s 3′ UTR length has a general negative correlation with its expression in six types of cancers (LUSC, LUAD, UCEC, BLCA, BRCA, and KIRC, see). We next examined in HCC tumor in Chinese patients and found a similar correlation between 3′ UTR length change and expression alteration at RNA level. However, the degree of 3′ UTR length change seems not always correlated well with gene expression alteration, as exemplified by sample 1 and 34 (Figure 1D and Figure 2B). We speculate that two possible reasons may explain this. The first one is the heterogeneity of HCC tumors. The tumor tissue from different patients may contain different percentage of contamination of normal cells, and the degree of infiltration of immune cells could also be different among samples. These heterogeneous cells may partially contribute to the weak correlation between 3′ UTR length changes and expression alteration. The second possible explanation is that alternative polyadenylation (APA) is not the only contributor to steady-state mRNA level of *HN1*. Transcriptional regulation and/or RNA degradation could also play a role, which deserves future study.

The downstream mechanism underlying *HN1*-KD induced senescence deserves further investigation. Interestingly, anti-HN1 pull-down coupled with mass spectrum showed high enrichment of ribosomal-related proteins (Supplementary Figure 17A), indicating that reduced *HN1* may contribute to senescence-associated phenotypes through translational level rather than RNA level. Considering that inhibition of ribosomal RNA (rRNA) biosynthesis by chemical inhibitor CX-5461 or other factors reduces global protein production and promotes senescence-associated phenotypes [42, 43], we examined whether *HN1*-KD induced senescence could be explained by reduced translation. Notably, decreased total protein levels and *HN1* expression were observed in CX-5461-treated HUVECs and A549 cells (Supplementary Figure 17B–17E). Moreover, the rRNA synthesis inhibitor CX-5461 could also induce the increased senescence-associated SA-β-Gal activity in both HUVEC and A549 cells (Supplementary Figure 17F–17I). Intriguingly, down-regulation of *HN1* with two different shRNAs both reduced total protein abundance (Supplementary Figure 17J). However, the translation-dependent mechanism of HN1 in explaining senescence required future study.

As an RNA binding protein, HNRNPA1 has been reported to interact with many different RNAs and be involved in multiple aspects of RNA metabolism. HNRNPA1 may interact with target RNA through a sequence-based way. The telomere RNA containing UUAGGG repeats can interact with HNRNPA1 to regulate the telomere length and function [44, 45]. HNRNPA1 could also bind to RNAs with other sequence motifs, such as the UAGA motif [46], AU-rich or GU-rich motif in the 3′ UTR [47]. Moreover, HNRNPA1 could also interact with RNA through a structure-based way. For example, it could bind to the G-quadruplex loops in the telomere RNA and in turn regulate telomere maintenance [48, 49]. Considering that telomere dysfunction is a hallmark of aging [50], the results above provide credible clues that HNRNPA1 may play a role in cellular aging. According to the iCLIP datasets in POSTAR2 [51], HNRNPA1 could potentially target to thousands of genes, suggesting that HNRNPA1 has the potential to function in various molecular and biological processes.

HNRNPA1 is a well-known splicing factor involved in the regulation of human global alternative splicing events [52]. Comprehensive misregulation of HNRNP proteins and alternative splicing disorders have been shown in many types of cancers [53]. Therefore, the role of *HNRNPA1* in cancers has been thoroughly studied. For example, *HNRNPA1* is highly expressed in hepatocellular carcinoma [54] and lung adenocarcinoma [55] to promote tumor progression. Down-regulation of *HNRNPA1* could suppress tumorigenesis by cell cycle arrest [55] or by inducing apoptosis in cancer cells [56]. Defects of *HNRNPA1* could also cause aging-related diseases, such as amyotrophic lateral sclerosis (ALS) [57] and Alzheimer’s disease [58]. In addition to the splicing-dependent function that may explain cancer-related phenotypes and other age-related diseases, the splicing-independent function of HNRNPA1 is also important for a well-understood regulatory mechanism. Indeed, previous studies have demonstrated multiple splicing-independent functions of HNRNPA1, such as transcription, mRNA transport, stability and translation [59]. Here, we identified HNRNPA1 as an important APA regulator of *HN1*.

Altogether, we provided evidence that HNRNPA1-mediated APA regulation of *HN1* can contribute to *HNRNPA1*-KD-induced senescence. Targeting the HNRNPA1-*HN1* axis may provide a new perspective for the potential treatment of cancer and other age-associated diseases.

## MATERIALS AND METHODS

### Cell culture and transfection

Human HEK293T, A549, and HUVEC cells were routinely cultured in Dulbecco's Modified Eagle Medium (DMEM, Gibico) supplemented with 10% (v/v) fetal bovine serum (FBS) in a CO_2_ incubator with 5% CO_2_ at 37 °C. Stable knockdown of *HN1* and *HNRNPA1* in these three cell lines was performed using lentiviral short hairpin RNA (shRNA) constructs, with plasmid pLKO.1 served as the control. The clone IDs of shRNA were obtained from Sigma-Aldrich as follows, shHN1_#1: TRCN0000140351; shHN1_#2: TRCN0000139615; shHN1_#3: TRCN0000143748; shHN1_#4: TRCN0000139039; shHNRNPA1_#1: TRCN0000235097; shHNRNPA1_#2: TRCN0000235098. Lentiviral vectors were constructed according to established protocols from the Broad Institute RNAi Consortium. Cells were transfected with lipofectamine 2000 (Invitrogen). Infected cells were then screened in DMEM supplemented with 2 μg/ml puromycin (Sigma-Aldrich).

### 3′ RACE and Sanger sequencing

Total RNA was extracted with TRIzol Reagent (Sigma) according to the manufacturer’s protocol. 500 ng RNAs were reversely transcribed into cDNAs with sequence-coupling oligo (dT), using the FastQuant RT kit (TianGen). PCR was performed with primers specific to given 3′ UTR of *HN1*. Then the 3′ RACE products were cloned into pGEM-T-easy vector and sequenced by Sanger’s method.

### Vector construction for overexpression and dual luciferase assay

To construct overexpression plasmids for *HN1* and *HNRNPA1*, cDNAs were amplified from HEK293T and cloned into pCDH vectors with HA tags or pCMV vector with FLAG tags. To test the efficiency of two transcripts of *HN1*, different 3′ UTR sequence was amplified from human genomic DNA and cloned into psiCHECK2 vector (Promega, cat. no. C8021) using the XhoI and PmeI restriction enzyme sites located at the 3′ end of the *Renilla* gene. All construct sequences were confirmed by Sanger sequencing. Primers used for cloning are listed in Supplementary Table S1.

### Dual luciferase reporter assay

Cells (HEK293T, A549, and HUVEC) in 24-well plate were transfected with luciferase reporter plasmids (psiCHECK2 and those inserted with long or short 3′ UTR of *HN1*) for four replicates and harvested after 24 hours. 100 μl lysis buffer was added to each well and the plate was shaken at room temperature for 15 minutes (min). Then the luciferase activities in the cell lysates were measured using the dual-luciferase reporter 1000 assay system (Promega) according to the standard protocol.

### Quantitative reverse transcription PCR

Total RNA was extracted by TRIzol Reagent (Sigma). cDNA synthesis was then carried out from 500 ng DNA-free total RNA using random hexamers and FastQuant RT kit (Tiangen). Triplicate samples were subjected to quantitative PCR analysis using SYBR Green (Vazyme) for *HN1*, *HNRNPA1*, and two isoforms of *HN1* with different 3′ UTR length (*HN1-S*, *HN1-L*). *GAPDH* served as an endogenous control. The information of these primers and senescent markers (*CDK1*, *CDK2*, *CCNB1*, *CCNE1*, *IL6*, *RB1*, and *p27*) were listed in Supplementary Table S1.

### Western blot

Total proteins were extracted by TRIzol Reagent (Sigma) according to vendor’s protocol and resolved in 5% SDS solution. 20 μg of total proteins were subjected to SDS-PAGE, then transferred to nitrocellulose membranes. Membranes were incubated with anti-HN1 (Rabbit mAb, Abcam, ab126705), anti-HNRNPA1 (Rabbit mAb, Abcam, ab177152), and anti-GAPDH (Mouse mAb, Abcam, ab9484) separately at room temperature for 2 hours (h), then washed with TBST buffer, and incubated with corresponding secondary antibodies conjugated with horseradish peroxidase (HRP) (Anti-rabbit IgG, Cell Signaling Technology, #7074; Anti-mouse IgG, Cell Signaling Technology, #7076) for 1 h. Then protein levels were detected by ECL (Tanon) and images were captured using an imaging system (SageCreation).

### mRNA stability assay

Cells were treated with 5 ng/ml of Actinomycin D (Act D, which can stop new transcription; Sigma-Aldrich, A4262) for 0, 2, 4, 6, 8 10, 12 and 24 h and harvested at each time point. RNA was extracted and reversely transcribed into cDNA. Transcripts with different 3′ UTR (*HN1-S*, *HN1-L*) were subjected for qRT-PCR to measure mRNA level of the corresponding isoform of *HN1* at each time point.

### RNA immunoprecipitation

48 hours after transfection of *HNRNPA1-*overexpression plasmid in 293T and HUVEC, cells were washed with ice-cold PBS and harvested into two Eppendorf tubes. The collected cells were then centrifuged and resuspended in an equal pellet volume of RIP buffer (150mM KCl, 25mM Tris pH 7.4, 5mM EDTA, 0.5mM DTT, and 0.5% NP-40) supplemented with fresh RNase inhibitor (Applied Biosystems, cat. no. N8080119) and protease inhibitor (Roche, cat. no.4693116001) and were put on ice for 10min. 20% of the supernatant was saved as RNA input and the remaining lysate was used for IP with 10 μg anti-HA antibody or negative control IgG at 4°C overnight. Protein G beads were washed three times with RIP buffer on a magnetic concentrator and then added to each IP sample to incubate with rotation for 2 hours at 4 °C. Then wash beads three times with cold RIP buffer and once with PBS. Afterward, the RNA-protein complexes were digested with proteinase K at 65 °C for 1 h. RNA was purified using TRIzol Reagent and analyzed by RT-PCR or qRT-RCR.

### SA-β-Gal staining

Cells were seeded into 24-well/12-well plate with about 60% cell confluence one day in advance. Then the standard procedure of SA-β-Gal staining kit (Sigma, Biovision, cat. no. K320-250) was performed. After removing the culture medium, cells were washed twice with PBS, then fixed for seven minutes in fixation solution (1X), followed by three times washes with PBS, then incubated at 37 °C overnight in the fresh-mixed staining buffer. The color stained images were captured under the microscope (Leica).

### Cell proliferation and EdU incorporation assays

Cell proliferation rate was assayed using Cell Counting Kit-8 (CCK-8) (Dojindo, Japan) and EdU incorporation assay was carried out using kFluor488 Click-iT EdU image kit (KeyGen BioTECH). For the CCK-8 assay, cells were seeded in a 96-well plate with 100 μl culture medium and at least 2,000 cells per well. Then 10 μl CCK-8 solution was added to each well, followed by 2-hour incubation, and the absorbance at 450 nm was measured using a microplate reader (TECAN). This measurement was performed every 24 hours and cell growth curve was drawn according to the absorbance value at each time point. For EdU incorporation assay, cells were seeded in a 24-well plate and incubated with 10 μM EdU for 2 hours. Changes in cell growth were assayed following the kFluor488 Click-iT EdU image kit instruction. Then images were captured by fluorescence microscope (Leica).

### Colony formation assay

For each well in a 6-well plate, 2 ml complete medium containing 200 cells was prepared. After culturing cells at 37 °C with 5% CO_2_ for 14 days, the supernatant was discarded and the plate was washed three times with PBS. The cells were then fixed with 4% paraformaldehyde for 10 min and stained with 1% crystal violet (Sangon) for 5min. Colonies were counted under a microscope.

### Cell migration assay

The 24-well transwell plate (8-μm pore size, Corning Life Sciences, USA) was used to perform cell migration assay according to the manufacturer's instruments. Briefly, cells (5 × 10^4^/well) were seeded into the upper chamber of a transwell filter with medium containing 1% FBS. Medium containing 10% FBS was added to the lower plate. After 72-hour or 18-hour incubation, cells migrated to the lower filter were fixed with 4% paraformaldehyde, washed with PBS, then stained with 1% crystal violet and counted under the microscope. Each experiment was repeated three times.

### Reactive oxygen species detection

The levels of intracellular reactive oxygen species (ROS) was measured by membrane-permeable probe 2′,7′-dichlorodihydrofluorescin diacetate (DCFH-DA) (KeyGen BioTECH), which can be hydrolyzed into non-fluorescent DCFH, and then oxidized by ROS to produce fluorescent 2′,7′-dichlorofluorescin (DCF). Briefly, HUVEC and A549 cells were seeded in a six-well plate, washed with serum-free medium, loaded with 10 μM DCFH-DA in serum-free DMEM. Following incubation in the dark at 37 °C for 30min, cells were harvested and fluorescence intensity was measured by flow cytometry (BD Bioscences).

### Cell cycle and cell death analysis

Cells were trypsinized, collected by centrifugating at 500 g for 5 min, washed twice with PBS, and then resuspended with PBS containing 0.03% Triton X-100 and 50 μg/ml Propidium Iodide (PI). Following incubation in tha dark for 10 min, cell cycle assay was performed using a BD Flow Cytometer. For cell death analysis, cell apoptosis was detected by fluorescein isothiocynate (FITC)-conjugated Annexin V and propidium iodide (PI) double staining according to manufacturers’ protocol (BD Bioscience). Treated cells were harvested, washed, and stained with 5 μl PI and 5 μl FITC-AnnexinV in the dark for 10min, followed by assessment performed in a BD Flow Cytometer. Both results were analyzed using ModFit and CellQuest Pro software respectively. Each sample was tested three times.

### Gene expression and survival analysis of TCGA datasets

The mRNA expression of *HN1* and *HNRNPA1* in many cancer types and the overall survival probability were measured using the TCGA datasets from GEPIA website [23].

### Human liver cancer sample collection

Hepatocellular carcinoma (HCC) samples and matched controls were obtained from Chinese HCC patients. All samples were collected with the informed consent of the patients and the experiments were approved by the ethics committee of Second Military Medical University. Clinical information was collected from patient records.

### Mass spectrometry analysis

The coding sequence of *HN1* was ligated to the pCMV-FLAG overexpression plasmid, and FLAG-tagged HN1 was prepared after transfecting the construct in HEK293T cells (8 x100 mm dishes). Two days later, cells were lysed in 0.1% NP-40 buffer and pCMV-FLAG-HN1 was immunopurified by anti-FLAG M2 agarose beads (Sigma), followed by SDS-PAGE and coomassie blue staining. The band corresponding to FLAG-HN1 was analyzed by liquid chromatography tandem mass spectrometry in the Proteomics platform of School of Life Science, Fudan University.

### Statistical analysis

Analysis was performed using GraphPad Prism software. Unpaired *t*-test was used to analyze the statistical significance of differences between the means of independent groups evaluated by qRT-PCR. Error bars reflect the standard error of the mean (SEM) for three replicates. Results are presented as mean ± SEM. * represents p < 0.05; ** represents p < 0.01; *** represents p < 0.001.

## Supplementary Material

Supplementary Figures

Supplementary Table
