# Supplementary material for: HNRNPA1-mediated 3′ UTR length changes of HN1 contributes to cancer- and senescence-associated phenotypes
Source: Aging (Albany NY). 2019 Jun 30;11(13):4407–37. doi: 10.18632/aging.102060 (PMC6660030; doi:10.18632/aging.102060)
Supplement: Supplementary Figures [file aging-11-102060-s002.pdf]

SUPPLEMENTARY FIGURES

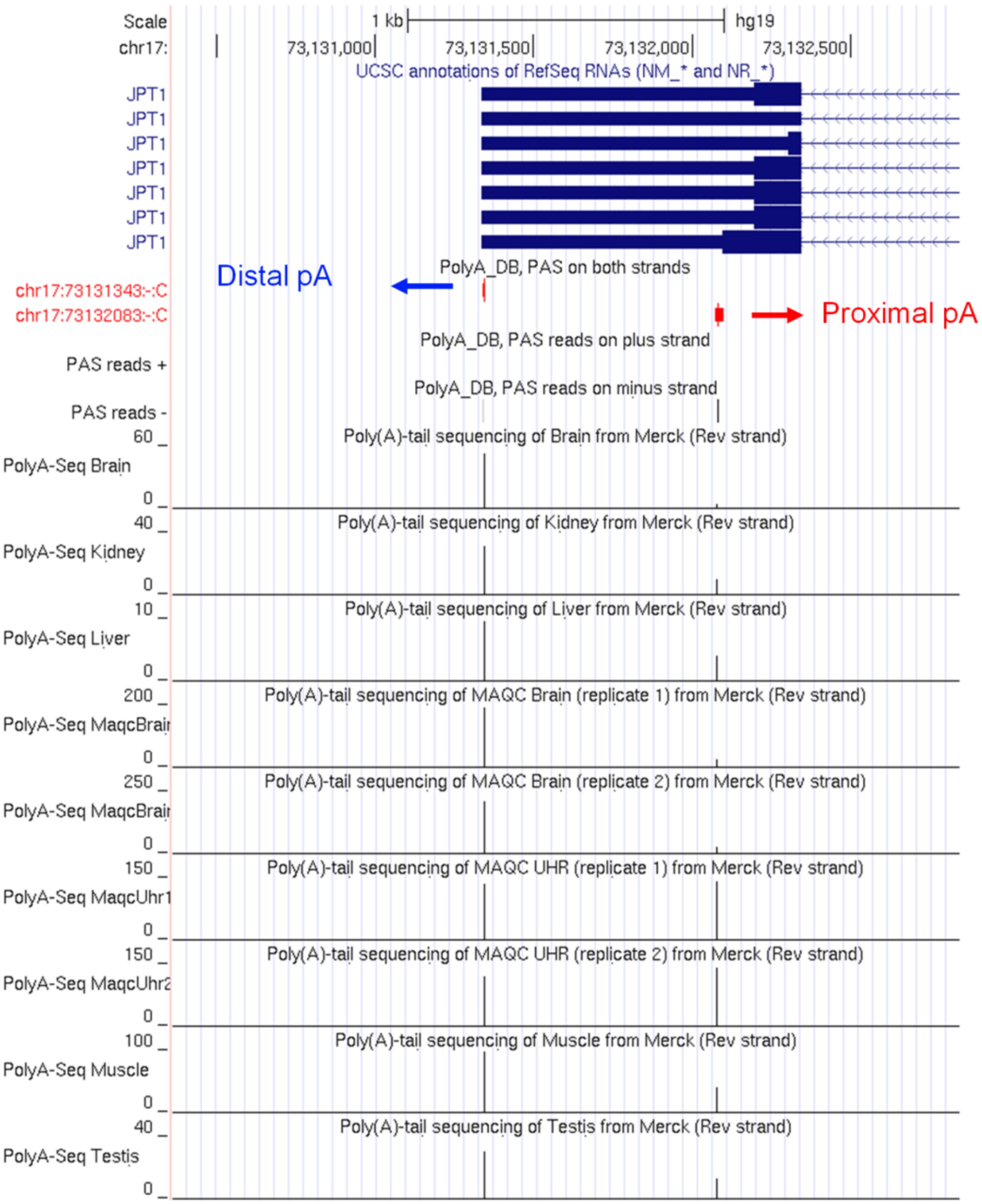

Supplementary Figure 1. Public PolyA-Seq tracks from multiple human tissues in UCSC genome browser shows signals of proximal and distal pA sites of *JPT1* (or *HN1*). RefSeq gene annotation and PolyA_DB3 tracks supported by 3′ READS were also shown in the browser with proximal pA site (Red) and distal pA site (Blue) of *HN1*.

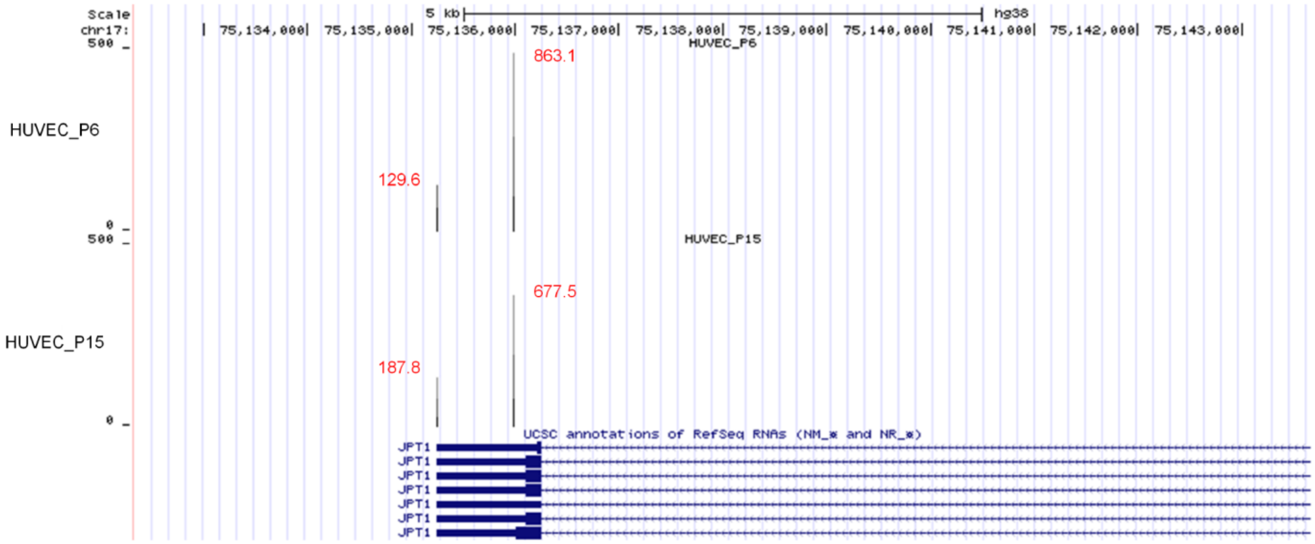

Supplementary Figure 2. Higher usage of distal pA site in senescent (passage 15 compared to passage 6) HUVECs, as illustrated by TPM (transcript per million) value at each pA site by PA-seq method [1].

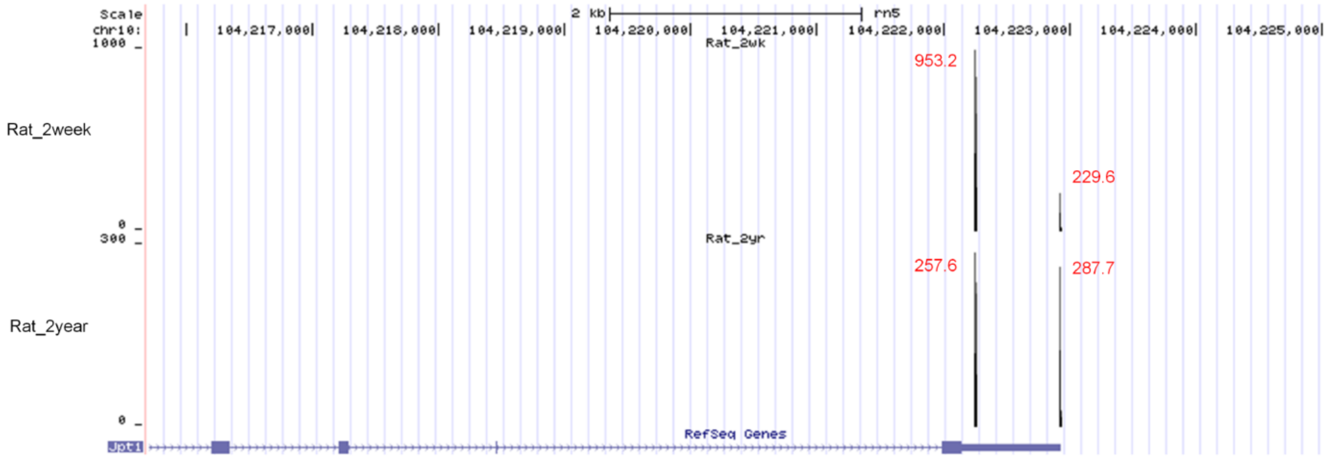

Supplementary Figure 3. Higher usage of distal pA site of *Hn1* in senescent rVSMC. TPM (transcript per million) value of each pA site by PA-seq method was shown.

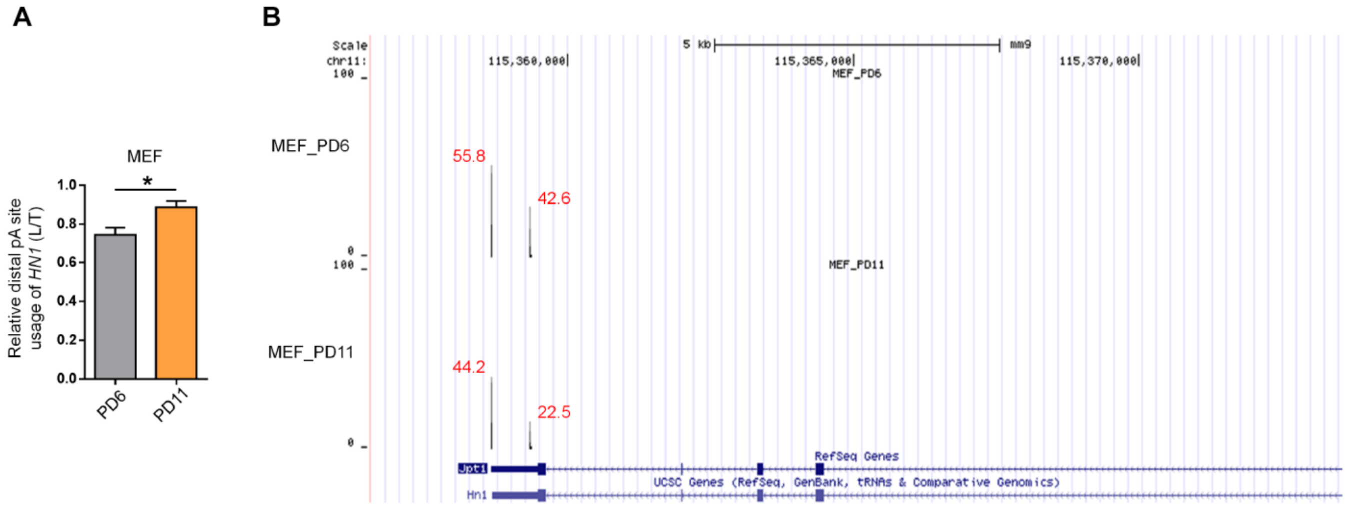

Supplementary Figure 4. Higher usage of distal pA site of *Hn1* in senescent MEFs. (**A**) The percentage of *Hn1-L* to total mRNAs (L/T) in different passages (PD11 compared to PD6) of MEFs was examined by qRT-PCR. (**B**) TPM (transcript per million) value of each pA site by PA-seq was shown.

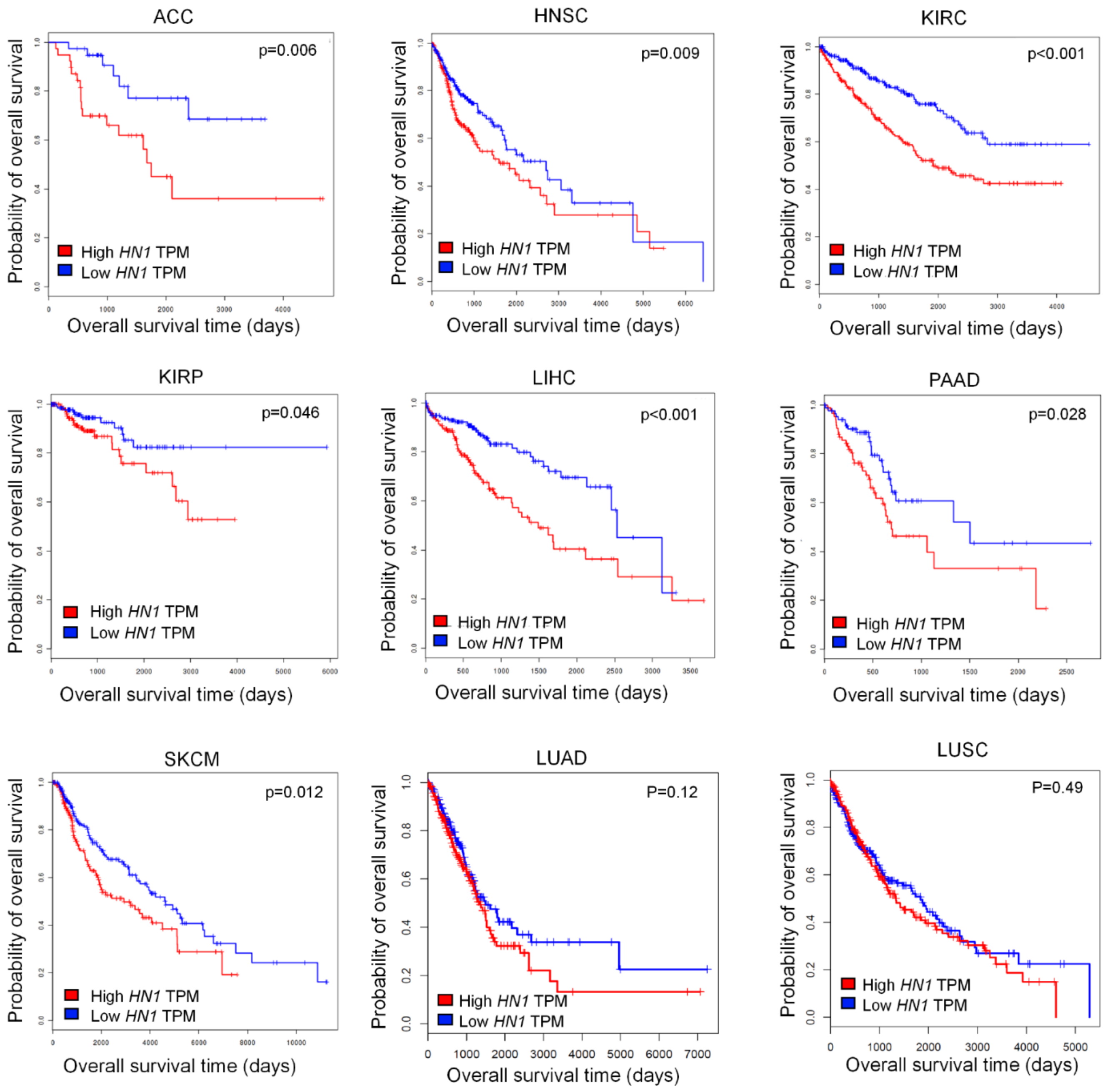

Supplementary Figure 5. Survival curves of patients with different expression of *HN1* in different types of cancer. Results from GEPIA based on TCGA data to show that patients with higher mRNA expression of *HN1* were associated with lower survival in nine cancer types (ACC, HNSC, KIRC, KIRP, LIHC, PAAD and SKCM, LUAD, and LUSC) [2].

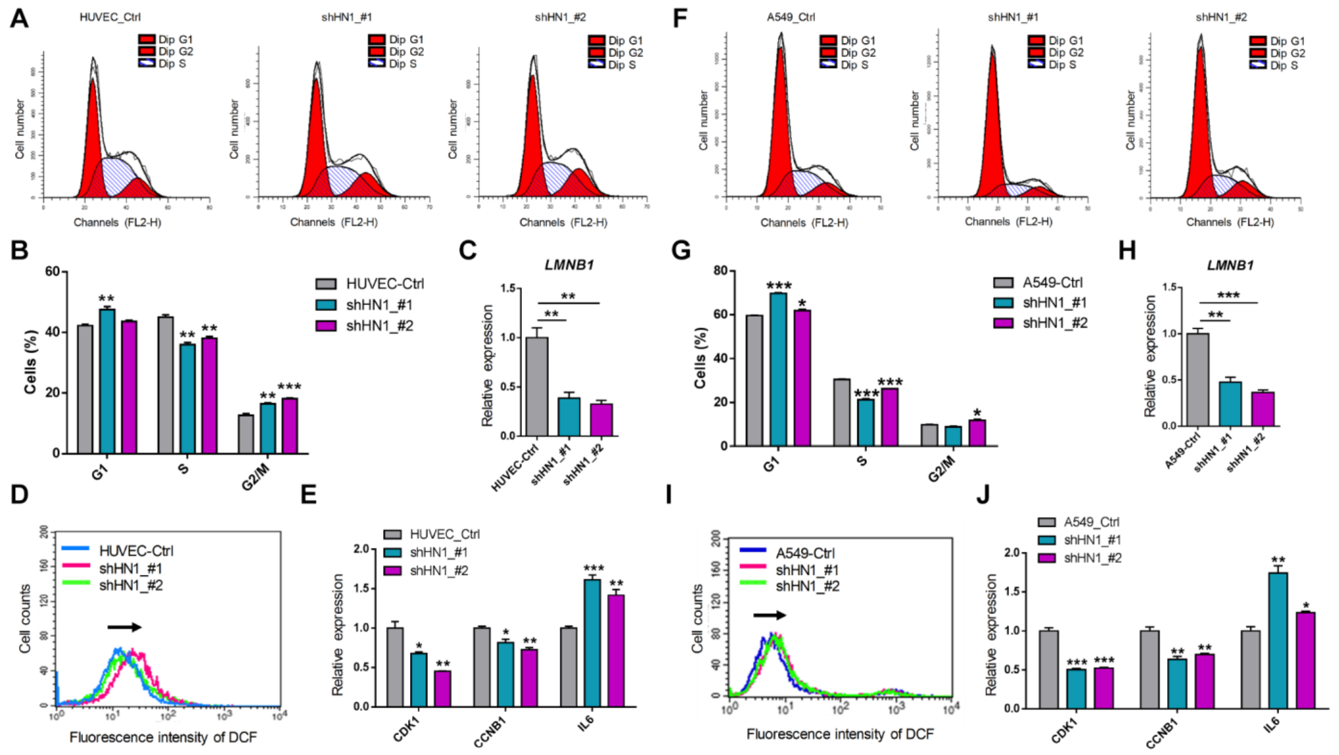

Supplementary Figure 6. Senescence-associated phenotypes in *HN1*-KD HUVEC and A549 cells. (**A**–**B**) Cell cycle arrest detected by Fluorescence-activated cell sorting (FACS) analysis (**A**) and corresponding quantitative evaluations (**B**) in *HN1*-KD and control HUVECs. (**C**) Decreased expression of *LMNB1* evaluated by qRT-PCR in *HN1*-KD HUVECs. (**D**) Reactive oxygen species (ROS) level measured by the fluorescence intensity of 2', 7'-dichlorofluorescin (DCF). (**E**) The mRNA expression level of *CDK1*, *CCNB1*, and *IL6* in *HN1*-KD HUVECs detected by qRT-PCR. (**F**–**G**) Cell cycle arrest detected by Fluorescence-activated cell sorting (FACS) analysis (**F**) and corresponding quantitative evaluations (**G**) in *HN1*-KD and control A549 cells. (**H**) Decreased expression of *LMNB1* evaluated by qRT-PCR in *HN1*-KD A549 cells. (**I**) ROS level measured by the fluorescence intensity DCF in A549 cells. (**J**) The mRNA expression level of *CDK1*, *CCNB1*, and *IL6* in *HN1*-KD A549 cells detected by qRT-PCR. *, ** and *** stand for $p < 0.05$, $p < 0.01$ and $p < 0.001$, respectively, based on *t*-test with three replicates.

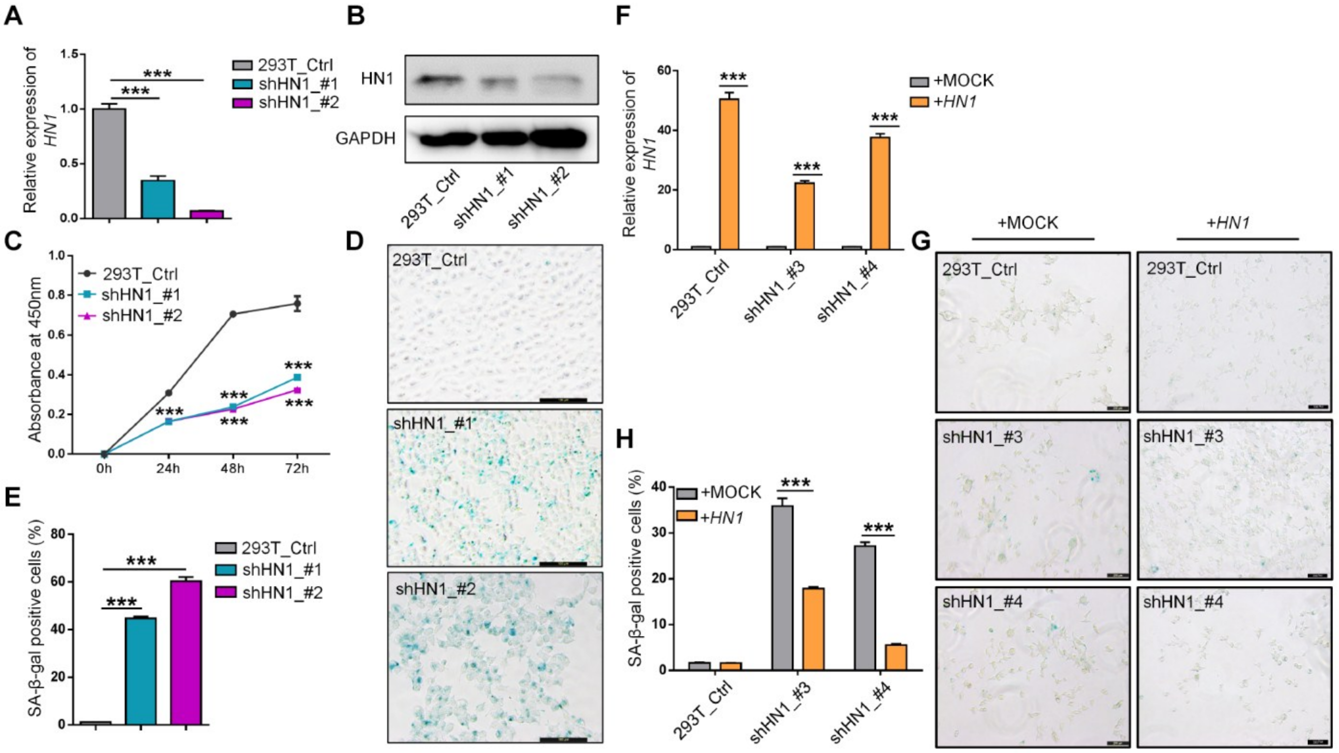

Supplementary Figure 7. Knockdown of *HN1* induced cellular senescence in HEK293T cells. (**A**–**B**) *HN1*-KD in HEK293T cells was validated by qRT-PCR (**A**) and Western blot (**B**). GAPDH served as a loading control. *** represents p < 0.001 based on *t*-test with three qPCR reactions. (**C**–**E**) CCK-8 assay (**C**) and SA-β-Gal staining (**D**, **E**) were performed upon *HN1* knockdown with two shRNAs. *** in C represents p < 0.001 based on *t*-test with three biological replicates. *** in E represents p < 0.001 based on *t*-test with three independent countings. (**F**–H) *HN1* overexpression reversed *HNRNPA1*-KD induced SA-β-Gal staining in HEK293T cells. *HN1* overexpression was confirmed in both *HNRNPA1*-KD and control HEK293T cells (**F**). *** represents p < 0.001 based on *t*-test with three qPCR reactions. Representative SA-β-Gal staining (**G**) and staining-positive cell statistics (**H**) in control (+MOCK) and overexpression (+*HN1*) HEK293T cells were shown. *** in H represents p < 0.001 based on *t*-test with three independent countings.

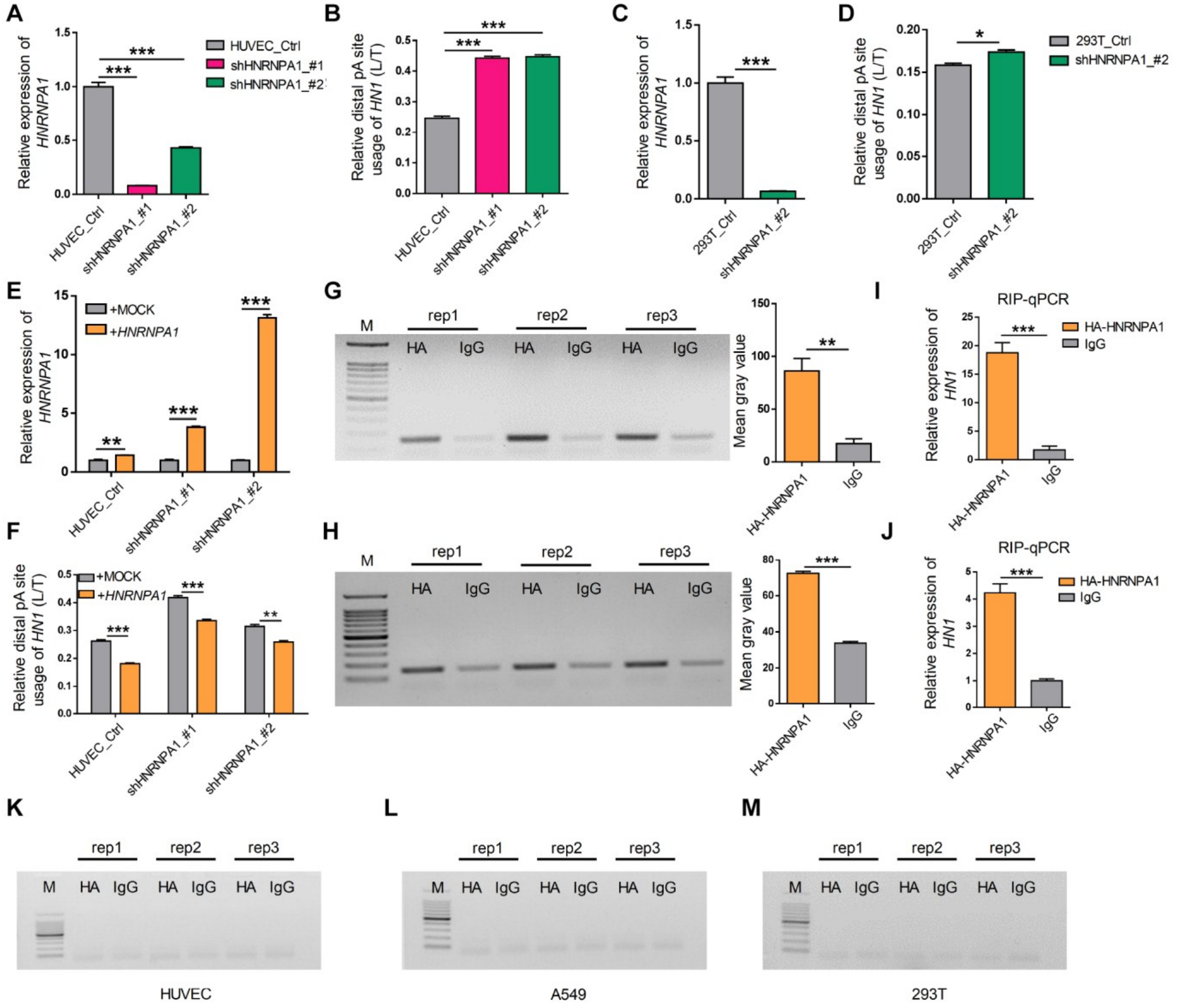

Supplementary Figure 8. HNRNPA1 binds to *HN1* mRNA and regulates its 3′ UTR length in HEK293T and HUVECs. (**A**) The expression of *HNRNPA1* was quantified by qRT-PCR in HUVEC cells transfected with two shRNAs targeting *HNRNPA1* (shHNRNPA1_#1, shHNRNPA1_#2). *** represents $p < 0.001$ based on *t*-test with three qPCR reactions. (**B**) Validation of 3′ UTR lengthening of *HN1* upon *HNRNPA1* knockdown by qRT-PCR analysis, as evaluated by the relative ratio between *HN1-L* and total mRNA in HUVEC. *** represents $p < 0.001$ based on *t*-test with three qPCR reactions. (**C**) The expression of *HNRNPA1* was quantified by qRT-PCR in HEK293T cells transfected with one shRNA. *** represents $p < 0.001$ based on *t*-test with three qPCR reactions. (**D**) qRT-PCR to test the relative expression of *HN1-L* upon *HNRNPA1*-KD in HEK293T cells. * represents $p < 0.05$ based on *t*-test with three qPCR reactions. (**E**–**F**) The relative expression of *HN1-L* was quantified by qRT-PCR when *HNRNPA1* was rescued in *HNRNPA1*-KD HUVECs. The level of *HNRNPA1* was restored as quantified by qRT-PCR (**E**). The ratio of *HN1-L* to total mRNA was reduced upon overexpression of *HNRNPA1* in *HNRNPA1*-KD cells (**F**). ** and *** in E-F represent $p < 0.01$ and $p < 0.001$, respectively, based on *t*-test with three qPCR reactions. (**G**, **H**) RIP-PCR was performed in HUVEC (**G**) and HEK293T (**H**) cells transfected with the HA-tagged *HNRNPA1*-overexpression plasmid. Immunoprecipitated RNA with either HA-antibody or control IgG was reversely transcribed and amplified with primer pairs specific for *HN1* mRNA. Agarose gel of three independent RIP-PCR experiments (rep1, 2, 3) was shown at left and mean gray value was shown at right. ** and *** at the right panels of G-H represent $p < 0.01$ and $p < 0.001$, respectively, based on *t*-test with three RIP-PCR reactions at the left panels. (**I**, **J**) RIP-qPCR was performed to test the enrichment of *HN1* mRNA in HA-antibody compared to control IgG in HUVEC (**I**) and HEK293T (**J**) cells. *** represents $p < 0.001$ based on *t*-test with three qPCR reactions. (**K**–**M**) The binding of HNRNPA1 to mRNA of *GAPDH* was detected by RIP-PCR with three repetitions in HUVEC (**K**), A549 (**L**), and 293T (**M**). HA represents hemagglutinin (HA) tagged HNRNPA1, IgG represents negative control.

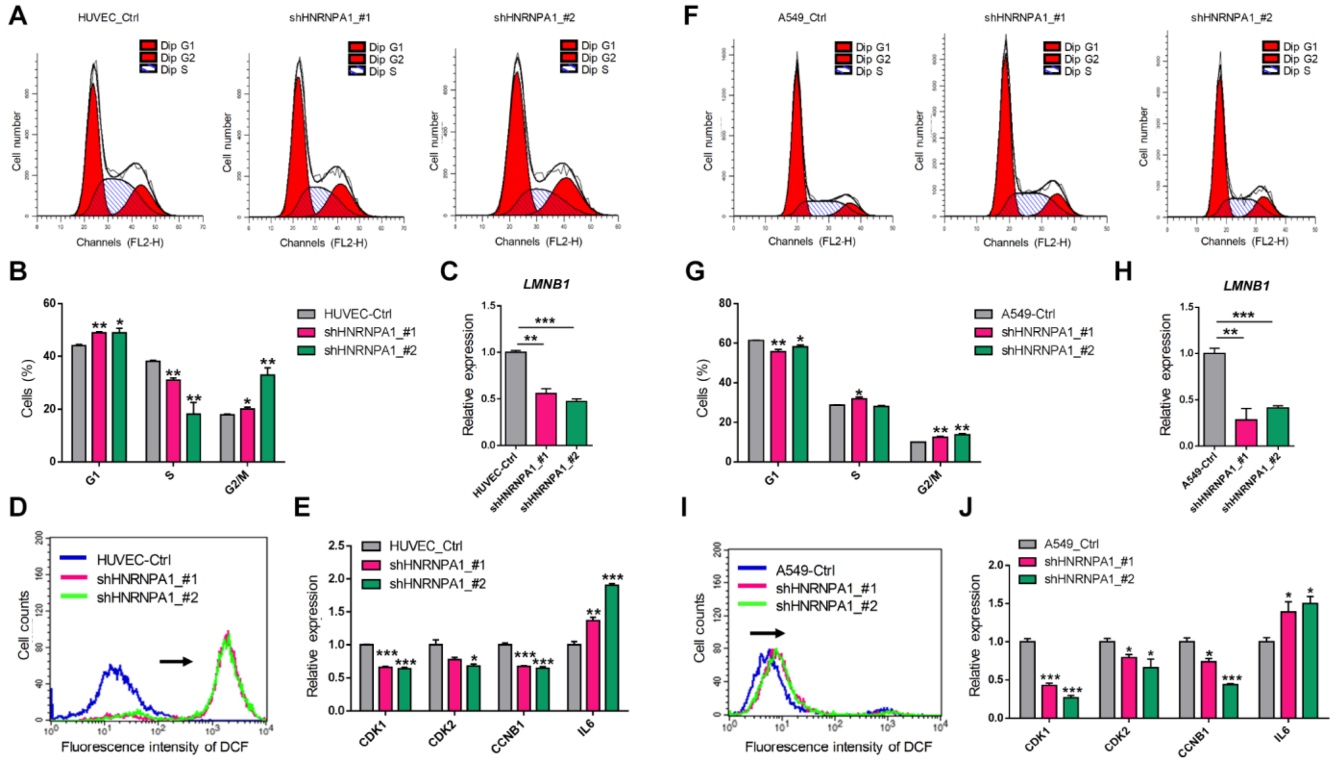

Supplementary Figure 9. Senescence-associated phenotypes in *HNRNPA1*-KD HUVEC and A549 cells. (**A**–**B**) Cell cycle arrest detected by Fluorescence-activated cell sorting (FACS) analysis (**A**) and corresponding quantitative evaluations (**B**) in *HNRNPA1*-KD and control HUVECs. (**C**) Decreased expression of *LMNB1* evaluated by qRT-PCR in *HN1*-KD HUVECs. (**D**) Reactive oxygen species (ROS) level measured by the fluorescence intensity of 2', 7'-dichlorofluorescin (DCF) in HUVECs. (**E**) The mRNA expression level of *CDK1*, *CDK2*, *CCNB1*, and *IL6* in HUVECs detected by qRT-PCR. (**F**–**G**) Cell cycle arrest detected by Fluorescence-activated cell sorting (FACS) analysis (**F**) and corresponding quantitative evaluations (**G**) in *HNRNPA1*-KD and control A549 cells. (**H**) Decreased expression of *LMNB1* evaluated by qRT-PCR in *HN1*-KD A549 cells. (**I**) ROS level measured by the fluorescence intensity DCF in A549 cells. (**J**) The mRNA expression level of *CDK1*, *CDK2*, *CCNB1*, and *IL6* in A549 cells detected by qRT-PCR. *, ** and *** stand for $p < 0.05$, $p < 0.01$ and $p < 0.001$, respectively, based on *t*-test with three replicates.

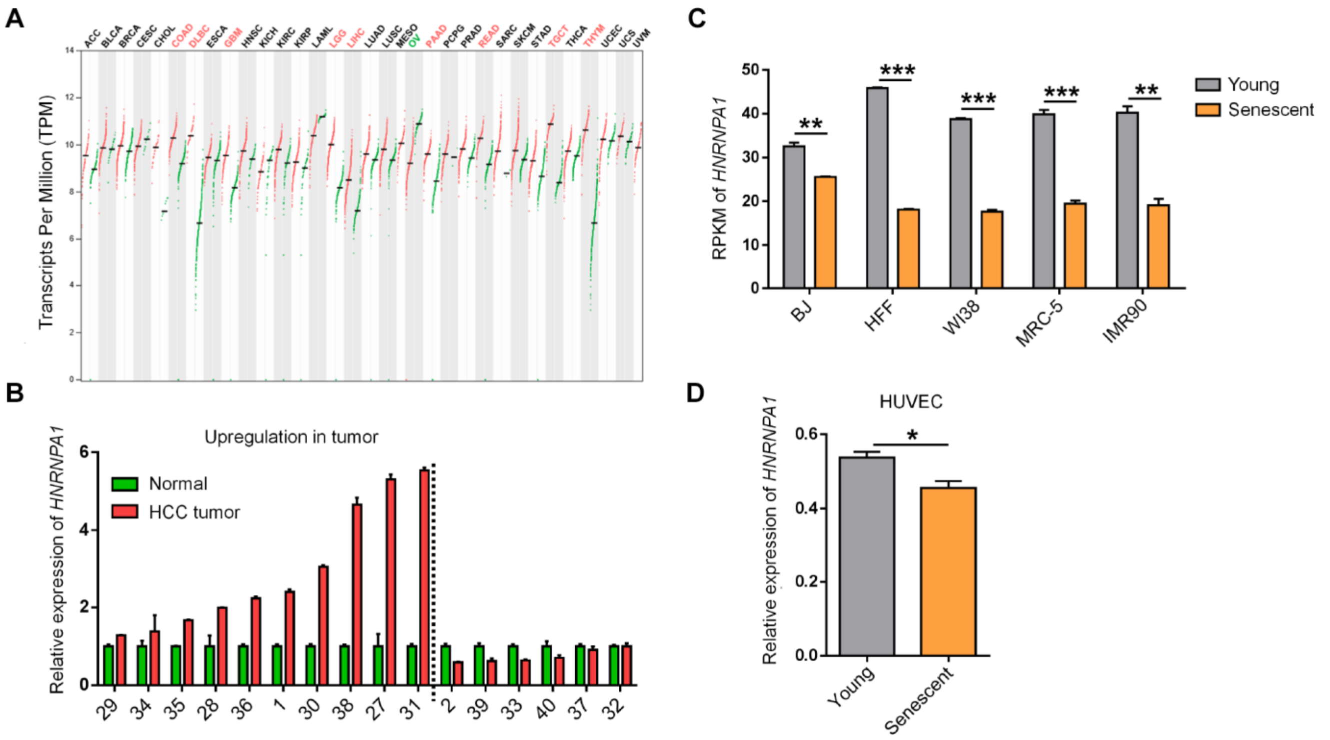

Supplementary Figure 10. The opposite expression pattern of *HNRNPA1* between cancer and senescence. (**A**) *HNRNPA1* mRNA expression in various cancer types based on TCGA datasets was obtained from GEPIA [2]. Significantly higher and lower mRNA levels of *HNRNPA1* when comparing tumor to matched normal tissue were shown as red and green fonts at the top, respectively. Each red line within the rectangle represents tumor tissues while the green line stands for normal tissues. Median TPM expression values were denoted as crossed black short line. (**B**) qRT-PCR assay to test total mRNA levels of *HNRNPA1* between normal and tumor tissues in 16 pairs of HCC patients. The number in X axis represents the same labeling ID of a patient as described in Figure 1D. Left of dashed line showed patients with upregulation of *HNRNPA1*. (**C**) The mRNA levels of *HNRNPA1* based on public RNA-seq datasets of various senescent cells. Lower expression was observed in five human senescent models, including BJ, human foreskin fibroblasts (HFF), WI-38, MRC-5 and IMR90. RPKM was used as an indicator of expression. ** and *** represent p < 0.01 and p < 0.001, respectively, based on *t*-test with three biological replicates. (**D**) Changes in *HNRNPA1* expression between young (passage 6) and senescent (passage 15) HUVECs were evaluated by qRT-PCR. * represents p < 0.05 based on *t*-test with three qPCR reactions.

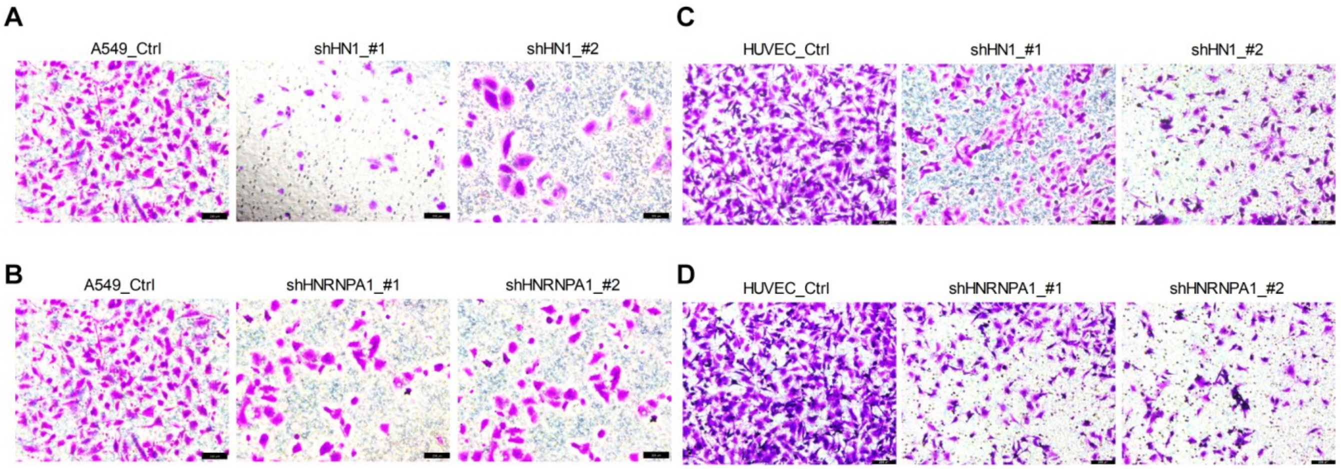

Supplementary Figure 11. Cell migration changes in HUVEC and A549 cells upon *HN1* and *HNRNPA1* knockdown at 18 hours after cell seeding. (**A**–**B**) Cell migration assay on *HN1*-KD (**A**) and *HNRNPA1*-KD (**B**) A549 cells. (**C**–**D**) Cell migration assay on *HN1*-KD (**C**) and *HNRNPA1*-KD (**D**) HUVECs.

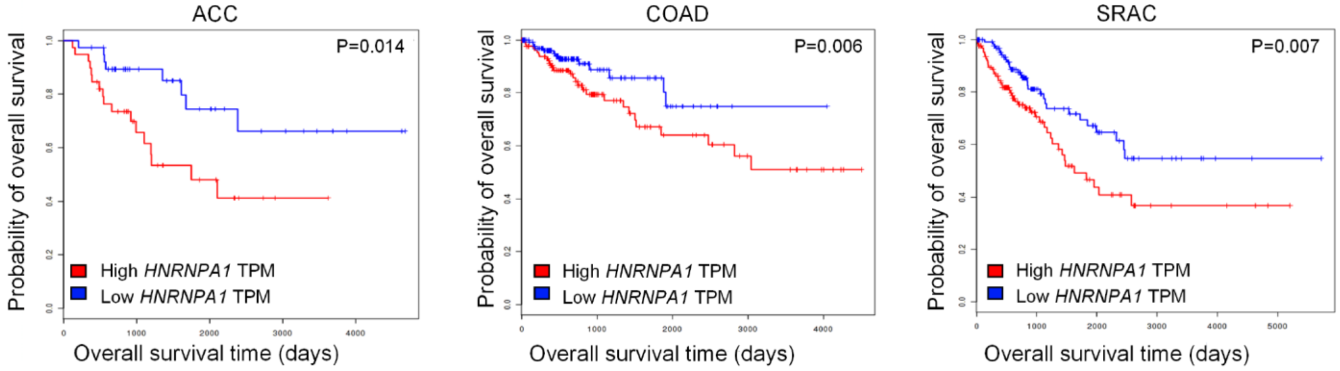

Supplementary Figure 12. High expression of *HNRNPA1* was associated with poor prognosis in cancer patients. Results from GEPIA [2] showed that patients with high expression of *HNRNPA1* in three cancer types (ACC, COAD, and SRAC) were associated with poor prognosis.

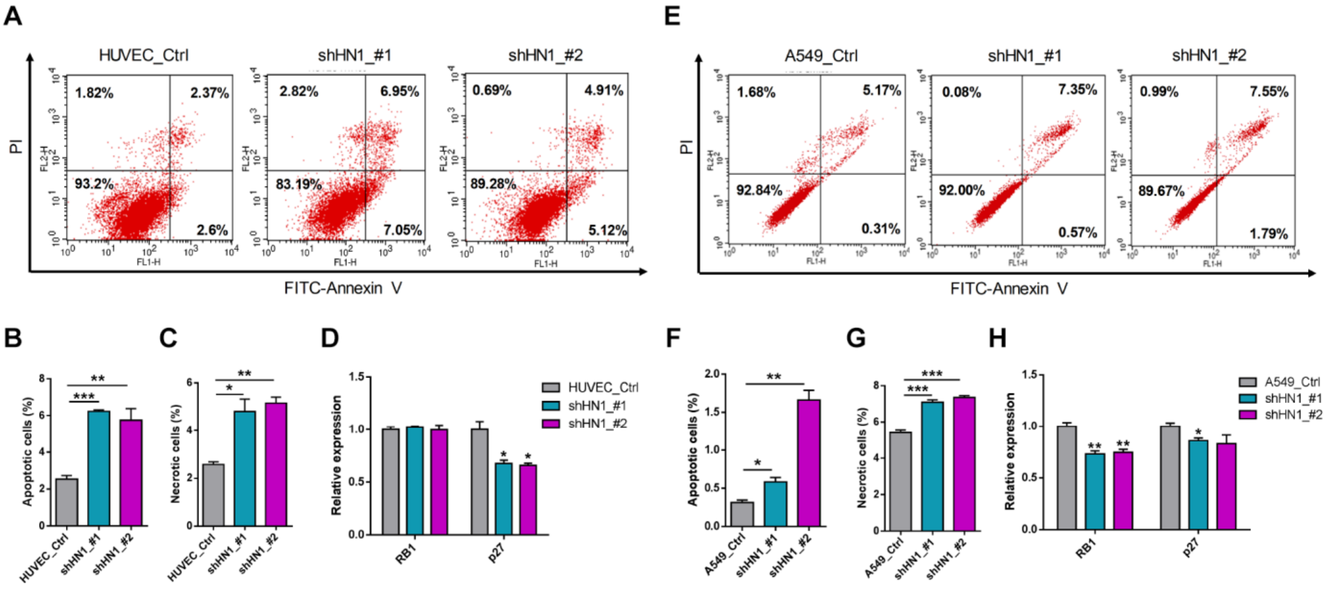

Supplementary Figure 13. *HN1*-KD induced apoptosis and necrosis in HUVECs and A549 cells. (**A**) Cell apoptosis and necrosis detected by fluorescein isothiocynate (FITC)-conjugated Annexin V and propidium iodide (PI) double staining and Fluorescence-activated cell sorting (FACS) analysis in HUVEC cells. (**B**–**C**) Quantitative evaluation for apoptotic cells (**B**) and necrotic cells (**C**) in panel A in *HN1*-KD and control HUVECs. (**D**) The mRNA expression of *RB1* and *p27* detected by qRT-PCR in HUVECs. (**E**) Cell apoptosis and necrosis detected by FITC-Annexin V and PI double staining and FACS analysis in A549 cells. (**F**–**G**) Quantitative evaluation for apoptotic cells (**F**) and necrotic cells (**G**) in panel E in *HN1*-KD and control A549 cells. (**H**) The mRNA expression of *RB1* and *p27* detected by qRT-PCR in A549 cells. shHN1_#1 and _#2 represent two shRNAs targeting *HN1* by lentivirus. *, ** and *** represent p < 0.05, p<0.01 and p < 0.001, respectively, based on *t*-test with three replicates.

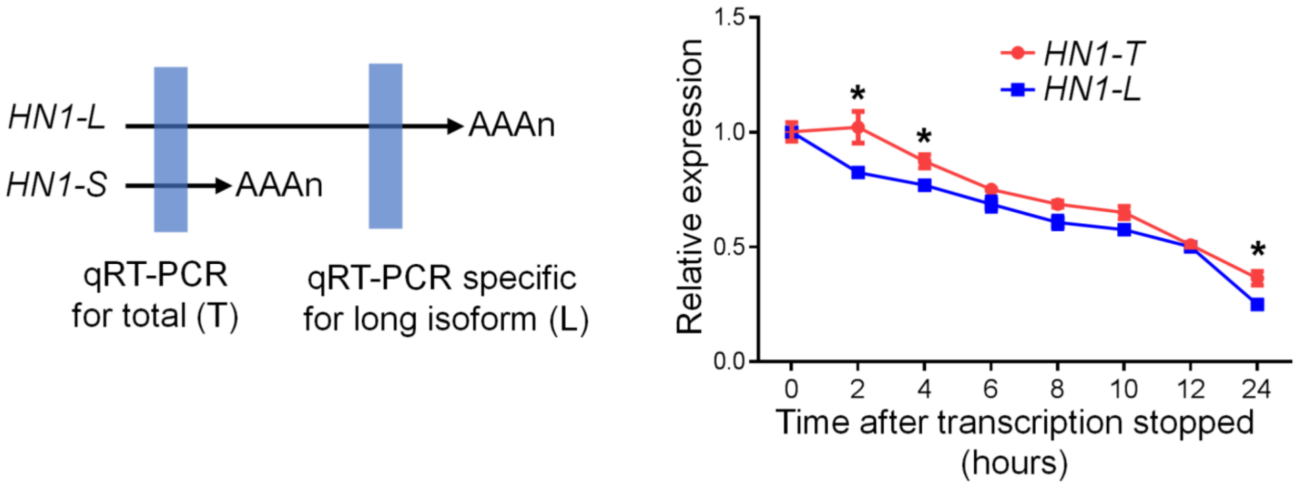

Supplementary Figure 14. RNA stability assay in HEK293T cells. The expression of long isoform (L) and total isoforms (T) was measured by qRT-PCR with specific primers (Left). Then the degradation rate of *HN1-T* and *HN1-L* was demonstrated by the relative expression at each different time points compared to 0 hours when blocking the transcription by Actinomycin D (Right).

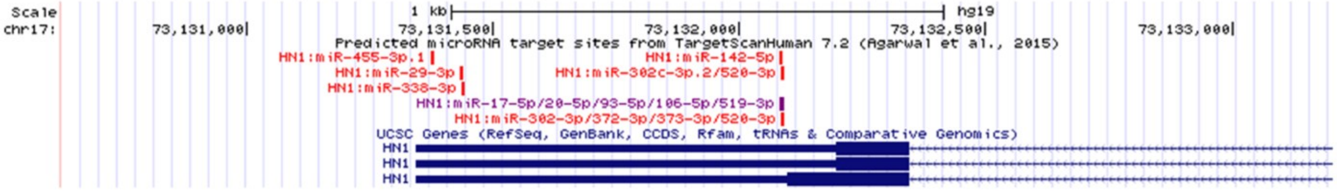

Supplementary Figure 15. The alternative 3′ UTR region of *HN1* has potential binding sites for regulatory microRNAs. Multiple conserved microRNAs (miR-455-3p, miR-29-3p and miR-338-3p) were predicted to bind to the alternative 3′ UTR region of *HN1* by TargetScan [3].

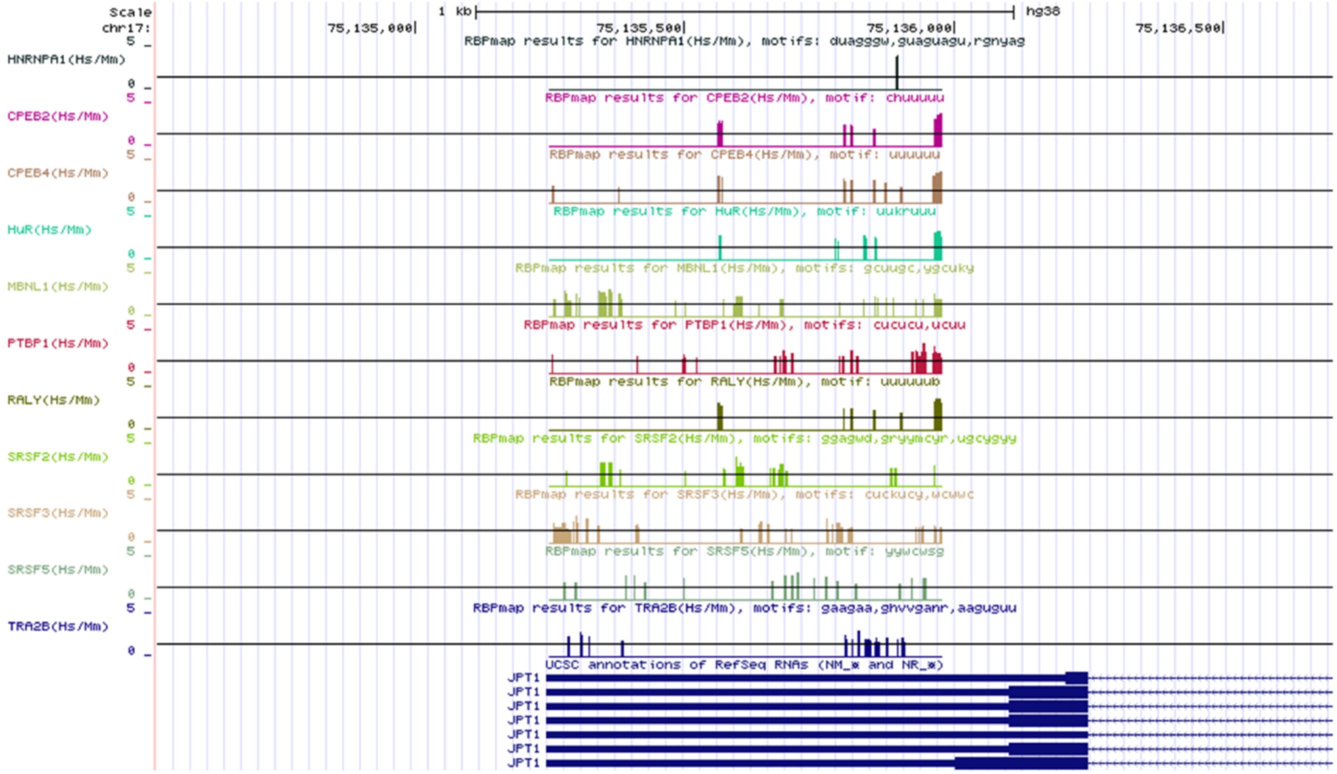

Supplementary Figure 16. The alternative 3′ UTR region of *HN1* has potential binding sites for regulatory RBPs. Multiple RNA binding proteins including HNRNPA1 were predicted to bind to the alternative 3′ UTR region of *HN1* by RBPmap [4].

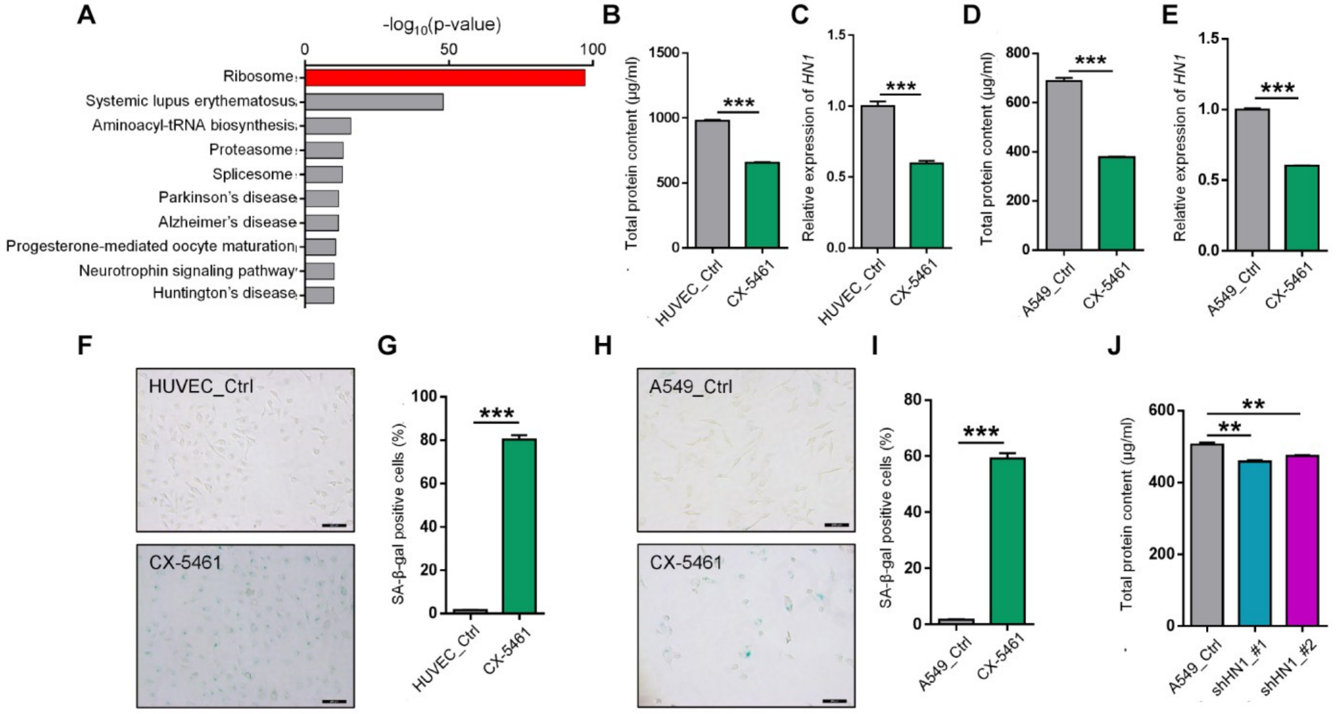

Supplementary Figure 17. Downregulation of *HN1* may contribute to rRNA biogenesis-related cellular senescence. (**A**) Mass spectrometry detection of proteins after *HN1* immunoprecipitation, followed by functional analysis using the Datasets for Annotation, Visualization and Integrated Discovery (DAVID) [5]. Top ten enriched pathways with the highest score in DAVID were shown. (**B**–**C**) Total protein levels (B) and *HN1* expression (**C**) detected after treatment with rRNA synthesis inhibitor, CX-5461, in HUVECs. *** represents $p < 0.001$ based on *t*-test with three BCA protein assays in B and three qPCR reactions in C. (**D**–**E**) Total protein levels (**D**) and *HN1* expression (**E**) detected after treatment with CX-5461 in A549 cells. *** represents $p < 0.001$ based on *t*-test with three BCA protein assays in D and three qPCR reactions in E. (**F**–**G**) SA-β-Gal staining (**F**) and quantitative statistics (**G**) in CX-5461 treated HUVECs. *** represents $p < 0.001$ based on *t*-test with three independent countings in G. (**H**–**I**) SA-β-Gal staining (**H**) and quantitative statistics (**I**) in CX-5461 treated A549 cells. *** represents $p < 0.001$ based on *t*-test with three independent countings in I. (**J**) Total protein levels detected by BCA protein assay in *HN1*-KD (two shRNAs) A549 cells. ** stands for $p < 0.01$ based on *t*-test with three replicates of BCA assay.

REFERENCES

1. Ni T, Majerciak V, Zheng ZM, Zhu J. PA-seq for Global Identification of RNA Polyadenylation Sites of Kaposi's Sarcoma-Associated Herpesvirus Transcripts. Curr Protoc Microbiol. 2016; 41:14e.7.1-e.7.8. https://doi.org/10.1002/cpmc.1 PMID:27153384

2. Tang Z, Li C, Kang B, Gao G, Li C, Zhang Z. GEPIA: a web server for cancer and normal gene expression profiling and interactive analyses. Nucleic Acids Res. 2017; 45:W98–102. https://doi.org/10.1093/nar/gkx247 PMID:28407145

3. Agarwal V, Bell GW, Nam JW, Bartel DP. Predicting effective microRNA target sites in mammalian mRNAs. eLife. 2015; 4:4. https://doi.org/10.7554/eLife.05005 PMID:26267216

4. Paz I, Kosti I, Ares M Jr, Cline M, Mandel-Gutfreund Y. RBPmap: a web server for mapping binding sites of RNA-binding proteins. Nucleic Acids Res. 2014; 42:W361-7. https://doi.org/10.1093/nar/gku406 PMID:24829458

5. Huang W, Sherman BT, Lempicki RA. Bioinformatics enrichment tools: paths toward the comprehensive functional analysis of large gene lists. Nucleic Acids Res. 2009; 37:1–13. https://doi.org/10.1093/nar/gkn923 PMID:19033363
