# Supplementary material for: HNRNPA1-mediated 3′ UTR length changes of HN1 contributes to cancer- and senescence-associated phenotypes
Source: Aging (Albany NY). 2019 Jun 30;11(13):4407–37. doi: 10.18632/aging.102060 (PMC6660030; doi:10.18632/aging.102060)
Supplement: Supplementary Table [file aging-11-102060-s001.pdf]

SUPPLEMENTARY TABLE

Supplementary Table 1. Primers information.

Primers	Sequence (5′ to 3′)
Hq_*HN1*_Forward	GTGAGGAAGAACAAAATGGCCT
Hq_*HN1*_Reverse	GGAGCTTGCTTCAGAGGAGT
Hq_*HNRNPA1*_Forward	TCTCCTAAAGAGCCCGAACA
Hq_*HNRNPA1*_Reverse	TTGCATTCATAGCTGCATCC
H_*HN1-S*_XhoI_Forward	CCGCTCGAGGACTGTCCTGAACGCTGTCG
H_*HN1-S*_PmeI_Reverse	GGGTTTAAACACAGCAGTACATAAAGTGCTT
H_*HN1-L*_XhoI_Forward	CTCGAGAAAGAAGCACTTTATGTACTGCTG
H_*HN1-L*_PmeI_Reverse	GTTTAAACTTCAAAATGTGGATATTGTTTACTTG
Hq_*HN1-S*_Forward	TGGGGCAGAGTGAAGAGAAG
Hq_*HN1-S*_Reverse	TGCAGTTCACAAGCATGGAG
Hq_*HN1-L*_Forward	CCTCCTCCTGTGACATGTGA
Hq_*HN1-L*_Reverse	TCAGTGTGATTGCCTCCCTT
H_*HN1* _Forward	GCTCTAGAATGACCACAACCACCACCTTC
H_*HN1* _Reverse	CGGAATTCATGAAACAAATCCAAGAGATGT
H_*HNRNPA1* _Forward	CGGAATTCATGTCTAAGTCAGAGTCTCCTAAAGAGC
H_*HNRNPA1* _HA_Reverse	CGGGATCCTACCCATACGATGTTCCAGATTACGCTAAATCTTCTGCCACTGCCATAG
Hq_*CDK1*_Forward	CCCTCCTGGTCAGTACATGG
Hq_*CDK1*_Reverse	GGCCAAAATCAGCCAGTTTA
Hq_*CDK2*_Forward	GTGGTACCGAGCTCCTGAAA
Hq_*CDK2*_Reverse	AAAGATCCGGAAGAGCTGGT
Hq_*CCNB1*_Forward	TGTGGATGCAGAAGATGGAG
Hq_*CCNB1*_Reverse	TGGCTCTCATGTTTCCAGTG
Hq_*CCNE1*_Forward	CCTCGGATTATTGCACCATC
Hq_*CCNE1*_Reverse	AGAATTGCTCGCATTTTTGG
Hq_*IL6*_Forward	CACAGACAGCCACTCACCTC
Hq_*IL6*_Reverse	TTTCAGCCATCTTTGGAAGG
Hq_*RB1*_Forward	TTGACACAACCCAGCAGTTC
Hq_*RB1*_Reverse	GCAACATGGGAGGTGAGAGT
Hq_*p27*_Forward	CCGGCTAACTCTGAGGACAC
Hq_*p27*_Reverse	CTTCTGAGGCCAGGCTTCTT
Hq_*GAPDH*_Forward	TGGAAGGACTCATGACCACA
Hq_*GAPDH*_Reverse	TTCAGCTCAGGGATGACCTT
